# Supplementary material for: Hebbian and Homeostatic Plasticity Mechanisms in Regular Spiking and Intrinsic Bursting Cells of Cortical Layer 5
Source: Neuron. 2015 Nov 4;88(3):539–52. doi: 10.1016/j.neuron.2015.09.025 (PMC4643308; doi:10.1016/j.neuron.2015.09.025)
Supplement: Document S1. Figures S1–S4 and Supplemental Experimental Procedures [file mmc1.pdf]

Neuron, Volume *88*

Supplemental Information

Hebbian and Homeostatic Plasticity Mechanisms in Regular Spiking and Intrinsic Bursting Cells of Cortical Layer 5

Stuart David Greenhill, Adam Ranson, and Kevin Fox

Figure S1

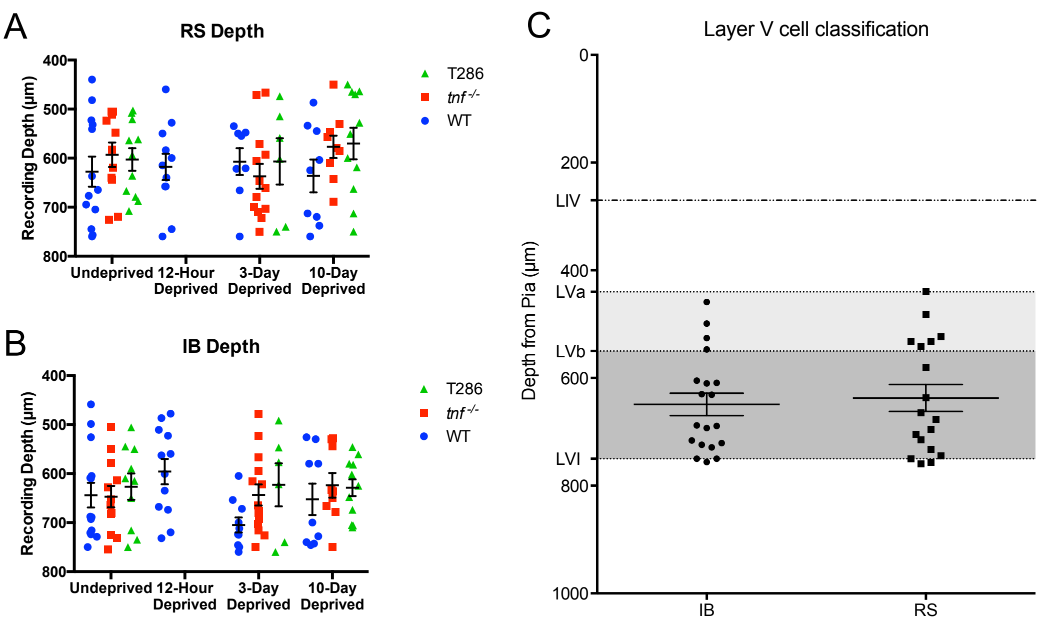

Figure S1 – Refers to Figure 1. Depth distribution of RS and IB cells recorded *in vivo* (left) and *in vitro* (right). **A** and **B.** No significant difference in depth was found between cohorts within cell types in the *in vivo* recordings (RS Deprivation: $F_{(2)}$ = 0.4219, P = 0.6572, RS Genotype: $F_{(2)}$ = 0.8028, P = s0.4516. IB Deprivation: $F_{(2)}$ = 0.6345, P = 0.5326, IB Genotype: $F_{(2)}$ = 2.063, P = 0.1333, 2-way ANOVA) either for the RS cells (top) or the IB cells (bottom). However, IB cells were encountered at a slightly greater depth than RS cells overall (644 ± 8.2 vs 607 ± 9.7, $t_{(185)}$ = 2.934, t-test). **C.** *In vitro* recordings showed that both RS and IB cell types were found throughout layer Va and Vb. No significant difference was found between the distributions of RS and IB cells in the cortical slice recordings ($t_{(35)}$ = 0.3675, mean $\pm$ sem = 688 $\pm$ 20 um for IB and 671 $\pm$ 25 um for RS, P = 0.7155, t-test). In all panels long horizontal lines indicate means and short horizontal lines indicate SEM

Figure S2

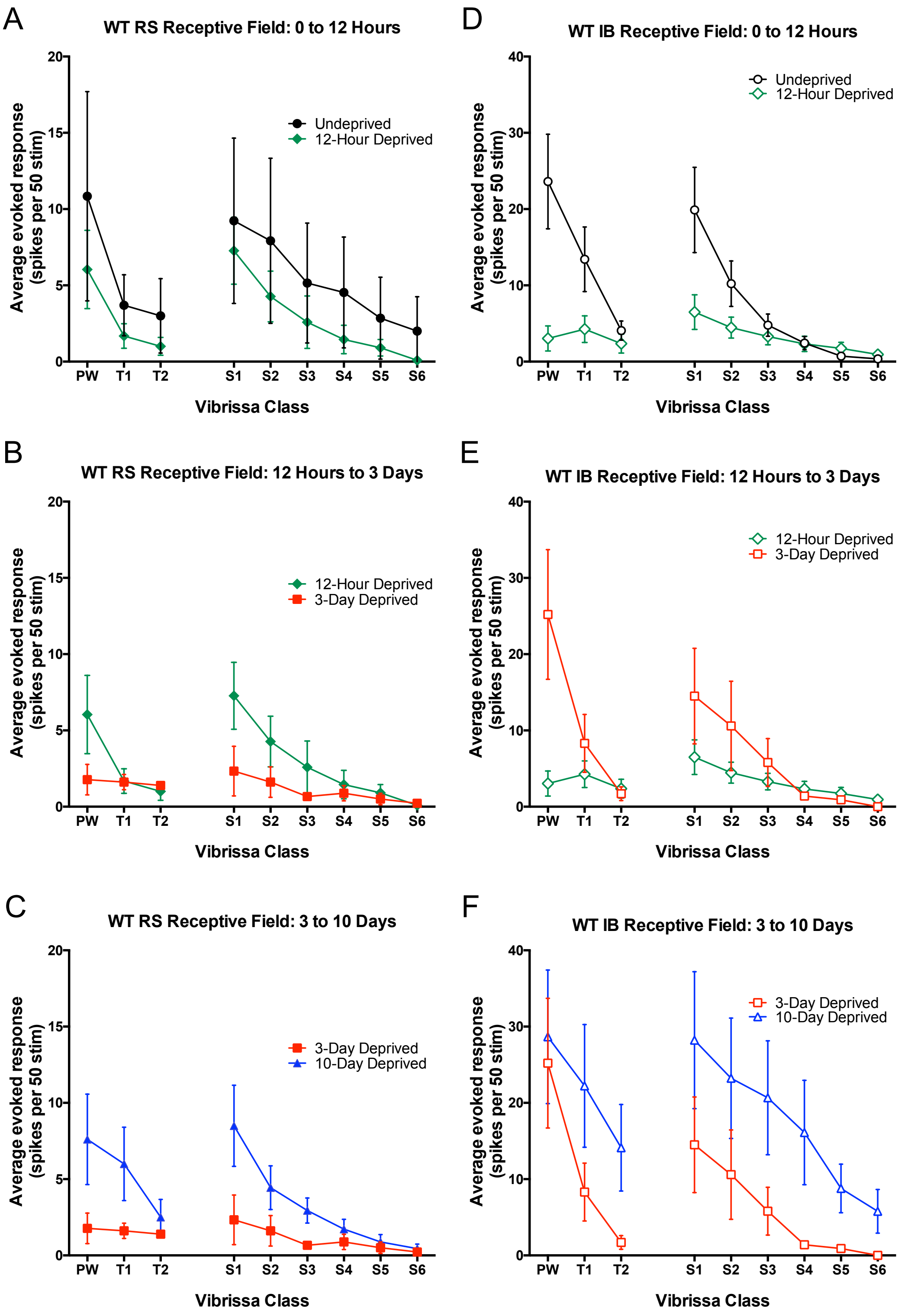

Figure S2 – Refers to Figure 2. Progression of spike responses in RS and IB cells from wild-type animals. **A.** RS cells show a slight depression between control and 12 hours deprivation (Mean response 5.47 ± 1.04 vs 2.81 ± 0.83

spikes per 50 stim) **B.** Further depression takes place between 12 hours and 3 days in RS cells (2.81 ± 0.83 vs 1.22 ± 0.23 spikes). **C.** By 10 days deprivation, RS cells have recovered towards baseline values (1.22 ± 0.23 vs 3.90 ± 0.97 spikes). **D.** IB cells show a more marked depression after 12 hours deprivation (8.83 ± 2.84 vs 3.21 ± 0.56 spikes per 50 stim). **E.** In sharp contrast to the RS cells, a recovery to baseline occurs in IB cells between 12 hours and 3 days deprivation (3.21 ± 0.56 vs 7.60 ± 2.75 spikes). **F.** IB cells, having already recovered by three days, display potentiation between 3 and 10 days deprivation (7.60 ± 2.75 vs 18.64 ± 2.69 spikes). Error bars represent SEM.

Figure S3

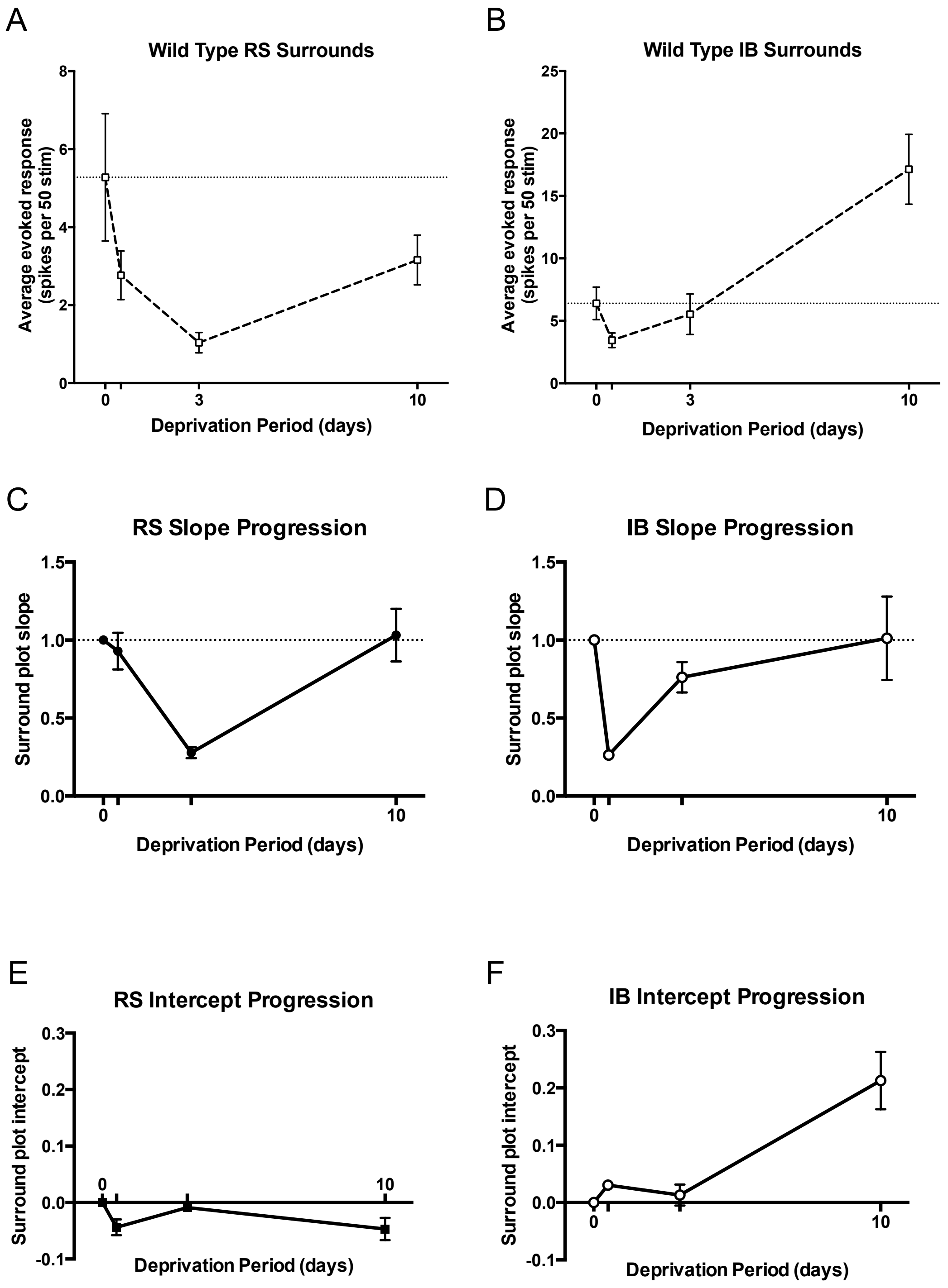

Figure S3 – Refers to Figure 3. The slope and intercept of surround responses can indicate multiplicative vs non-multiplicative plasticity shifts. **A.** Surround responses vs deprivation time in RS cells from wild-type mice. A slow depression to 3 days is followed by a partial recovery after 10 days (see also Figures 2, 3 and S2). **B.** IB surround responses show a fast depression at 12 hours, followed by recovery at 3 days and strong potentiation after 10

days deprivation (see also Figures 2, 3 and S2). **C.** The slope of the surround whisker plots displays a very similar progression to the spike responses in RS cells. A strong depression in slope at 3 days (0.28 ± 0.04 vs 1.0 for control) is followed by a return to baseline at 10 days (1.03 ± 0.17). This suggests that the control and 10 day surround receptive fields are similar but have undergone a non-multiplicative downward shift. **D.** Slope values for IB cells closely mimic the first part of the spike responses but not the latter. A rapid depression from control to 12 hours and rapid recovery to 3 days (1.0 to 0.26 ± 0.03 to 0.76 ± 0.1) is followed by a slow return to baseline as opposed to a potentiation (1.01 ± 0.27) after 10 days deprivation. **E.** RS intercepts display small downward shifts at 12 hours (-0.044 ± 0.013 12 hours, -0.047 ± 0.019 10 days). **F.** In contrast, the intercept of the IB surround plot exhibits a large positive shift between 3 and 10 days (0.013 ± 0.018 to 0.213 ± 0.050) suggesting that the potentiation seen in these cells is non-multiplicative. Error bars represent SEM.

Figure S4

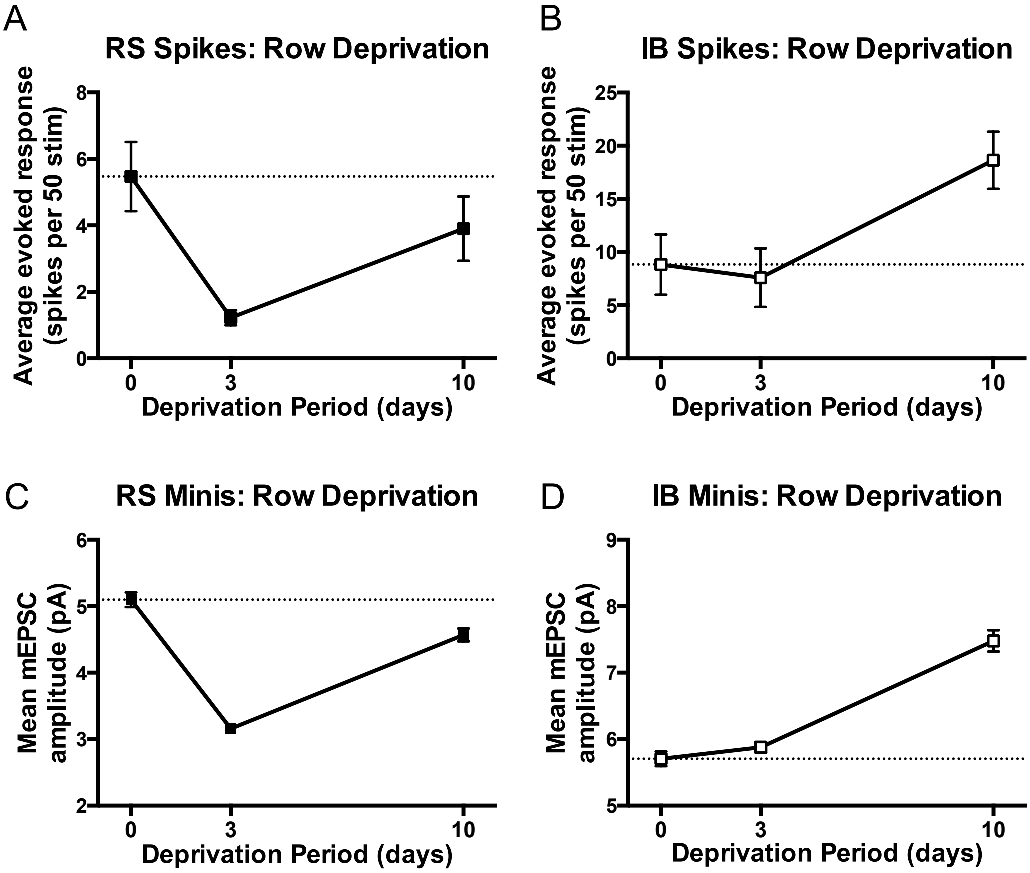

Figure S4 – Refers to Figure 5. Miniature EPSCs in row-deprived preparations *in vitro* display strong similarities to spiking responses *in vivo*. **A.** Summary of RS surround receptive field evoked spike responses – depression is followed by recovery. **B.** IB spikes, in contrast, show a strong potentiation between 3 and 10 days. **C.** RS cells in a D-row deprived *ex vivo* slice preparation: recording mEPSCs from the D-row barrels produces a progression in average mEPSC amplitude strikingly similar to that shown by the average surround receptive field spike responses. Depression at 3 days (5.10 ± 0.11 pA control vs 3.15 ± 0.04 pA 3 days) is followed by a recovery towards baseline (4.56 ± 0.10 pA) after 10 days deprivation. **D.** In contrast, the average mEPSC amplitudes of IB cells, much like their spike responses, show no depression at 3 days deprivation (5.71 ± 0.11 pA control vs 5.87 ± 0.08 pA at 3 days) but strongly potentiate after 10 days deprivation (7.48 ± 0.16 pA). Error bars represent SEM.

Supplemental Experimental Procedures

Subjects and Whisker Deprivation

All procedures were approved under the UK Animals (Scientific Procedures) Act 1986. All mice were either WT Jackson C57Bl/6J (Charles River, UK) or backcrossed into the Jackson C57Bl/6J background. A total of 63 adult male and female mice (27 C57Bl/6J wild-types, 20 TNF-α knockout (*tnf*$^{-/-}$) and 16 CamKII-T286A (T286), 6-10 weeks at recording, 213 cells total) were used for *in vivo* intracellular recording, with a further 10 wild-type mice used for histology *in vivo*. Thirty-one C57Bl/6J mice were used for *in vitro* recordings.

Subjects were lightly anaesthetised with isoflurane and had their entire right-side D-row trimmed to within 1mm of the vibrissal pad every 24-48 hours. Care was taken to trim the whiskers level with the surrounding fur but to leave enough stump for easy reattachment preceding the recording session. Deprived whiskers were replaced for recording by the corresponding whiskers from the contralateral side, attached with cyanoacrylate glue. Undeprived mice had their D-row whiskers acutely trimmed and re-attached immediately before the experiment.

In Vivo Recordings

Anaesthesia was induced with isoflurane and maintained by intraperitoneal injection of urethane (1.0 g/kg body weight) and a trace amount of acepromazine. Depth of anaesthesia was monitored by breathing rate and observation of reflexes, with supplementary urethane (0.1 g/kg) administered as necessary throughout the experiment. Body temperature was maintained at 37ºC using a thermostatically controlled heating blanket.

The location of the D-row was pinpointed before recording by intrinsic signal imaging with 700 nm light. Signals were acquired with an Optical Imaging 3001 ISI system and analysed with custom MATLAB code. Stimulus was presented as the rapid deflection of a single whisker at 5 Hz every 8 seconds using a piezoelectric wafer. At least two of D1, D2 or D3 barrels were identified and overlaid on to blood vessel patterns to gain the position and

orientation of the D-row. During electrophysiological recordings the position of the electrode was confirmed by the response to stimulus in layer IV.

After imaging a small (0.5-1 mm) craniotomy was made over the approximate location of the D2 barrel. A 30G hypodermic needle was used to open a small fleck of skull at the bottom of the craniotomy and then to resect the dura mater. A secondary craniotomy was made in posterior parietal cranium to allow for the placement of a carbon-fibre ground electrode. Borosilicate glass microelectrodes filled with 1M potassium acetate (50-120 MΩ) were passed through the small dura resection and the craniotomy covered with agar for stability. Recordings were performed with an Axoclamp 2B in current clamp mode with a manually balanced bridge and the electrode capacitance compensated. Data were acquired with a CED Micro-1401 digitiser and Spike2 software. IB and RS cells were distinguished by their response to injected current, with IB cells displaying a characteristic burst shape in response to depolarising current (Connors and Gutnick, 1990).

Whiskers were deflected using a 3x3 matrix of computer-controlled piezoelectric actuators in a custom-designed frame allowing for the resting position of the piezo to mimic the resting vibrissae position (Jacob et al., 2010; 2012). Piezo movement was controlled by a 9 whisker stimulator (CED 3901) and each piezo was regularly calibrated with a laser displacement measuring system (Micro-Epsilon). Receptive fields were quickly mapped during recording by a pseudorandom sequence of sparse noise ventrodorsal stimulations at 5Hz arranged in blocks of 10 (one of each vibrissae plus a non-stimulation event). Fifty blocks of stimulus were used for most cells, although between 15 and 100 sequences were used depending on the recording stability. Each whisker deflection was a trapezoidal stimulus (10ms rise, 10ms plateau, 10ms fall) of 300μm amplitude.

Analysis of In Vivo Recordings

Data were analysed using Spike2 software (CED) and custom R scripts. Action potentials were counted between 3-53 ms after each stimulation, with the count from the blank stimulus field subtracted as a measure of

background activity. To generate subthreshold waveforms, spikes were removed using the Wavemark tool of Spike2 and subtracting the generated spike waveforms from the original recording (Jacob et al., 2012). Whiskers were sorted from PW-T2 and S1-S6 based on their spike count alone. Subthreshold slope and latency were calculated as previously described (Jacob et al., 2012).

In Vitro Recordings

Mice (4-6 weeks old) were killed by cervical dislocation and decapitated, with the brain quickly removed and cooled in ice-cold dissection buffer (in mM: 108 choline-Cl, 3 KCl, 26 NaHCO3, 1.25 NaH2, PO4, 25 D-glucose, 3 Na-pyruvate, 1 CaCl2, 6 MgSO4, 285 mOsm, bubbled with 95% O_2 5% CO_2). Coronal slices (350μm) for depth analysis, or angled slices across the barrel rows (Finnerty et al., 1999) were cut on a vibrating microtome (Microm HM650V) and transferred to a holding chamber containing normal ACSF (in mM: 119 NaCl, 3.5 KCl, 1 NaH2PO4, 10 D-glucose, 2 CaCl2, 1 MgSO4, 300 mOsm bubbled with 95% O_2 5% CO_2). Slices were incubated at 32ºC for 45 minutes, then returned to room temperature before recording. Barrels were identified in slices under brightfield illumination and cells identified using differential interference contrast on an Olympus BX50WI microscope. Layer V was identified by distinct cellular morphology and density and whole-cell recordings were made from randomly-chosen neurones throughout both layer Va and layer Vb using borosilicate glass electrodes (4-8 MΩ) filled with a potassium-gluconate based solution (in mM: 110 K-gluconate, 10 KCl, 2 $MgCl_2$, 2 Na_2ATP, 0.03 Na_2GTP, 10 HEPES, 0.5% Biocytin, pH 7.3, 270 mOsm). For mini recordings the D-row barrel was visually identified and recordings made from within this barrel with the addition of 1μM tetrodotoxin, 10μM picrotoxin and 50μM AP-V. Recordings were made using an Axon Multiclamp 700B, digitized with a CED Micro 1401 and controlled with CED Signal software. Miniature EPSCs were analysed with Axograph software using a template-matching method.

After recordings the distance between the recording electrode and the pia was measured with Scientifica LinLab manipulator software and confirmed with a visual measurement on the microscope.

References:

Connors, B.W., and Gutnick, M.J. (1990). Intrinsic firing patterns of diverse neocortical neurons. Trends Neurosci. *13*, 99–104.

Finnerty, G.T., Roberts, L.S., and Connors, B.W. (1999). Sensory experience modifies the short-term dynamics of neocortical synapses. Nature *400*, 367–371.

Jacob, V., Estebanez, L., Le Cam, J., Tiercelin, J.-Y., Parra, P., Parésys, G., and Shulz, D.E. (2010). The Matrix: a new tool for probing the whisker-to-barrel system with natural stimuli. J. Neurosci. Methods *189*, 65–74.

Jacob, V., Petreanu, L., Wright, N., Svoboda, K., and Fox, K. (2012). Regular spiking and intrinsic bursting pyramidal cells show orthogonal forms of experience-dependent plasticity in layer V of barrel cortex. Neuron *73*, 391–404.
