# Supplementary material for: Hebbian and Homeostatic Plasticity Mechanisms in Regular Spiking and Intrinsic Bursting Cells of Cortical Layer 5
Source: Neuron. 2015 Nov 4;88(3):539–52. doi: 10.1016/j.neuron.2015.09.025 (PMC4643308; doi:10.1016/j.neuron.2015.09.025)
Supplement: Document S2. Article plus Supplemental Information [file mmc2.pdf]

Neuron

Article

Hebbian and Homeostatic Plasticity Mechanisms in Regular Spiking and Intrinsic Bursting Cells of Cortical Layer 5

Highlights

- RS and IB cells exhibit TNFα-dependent homeostatic recovery from depression
- IB cells exhibit CaMKII-dependent, input-specific potentiation, but RS cells do not
- TNFα-dependent homeostatic plasticity persists into adulthood in cortical layer 5
- mEPSCs of RS and IB cells mirror changes in their sensory evoked spike firing rates

Authors

Stuart David Greenhill, Adam Ranson, Kevin Fox

Correspondence

FoxKD@cardiff.ac.uk

In Brief

Using in vivo electrophysiology, Greenhill et al. show that layer 5 cell subtypes (regular spiking and intrinsic bursting) in the mouse barrel cortex show differing forms of plasticity that rely on different molecular mechanisms in response to sensory deprivation.

CrossMark

Greenhill et al., 2015, Neuron *88*, 539–552
November 4, 2015 ©2015 The Authors
http://dx.doi.org/10.1016/j.neuron.2015.09.025

CellPress

CellPress

Hebbian and Homeostatic Plasticity Mechanisms in Regular Spiking and Intrinsic Bursting Cells of Cortical Layer 5

Stuart David Greenhill,[1] Adam Ranson,[1] and Kevin Fox[1,*]
[1]School of Biosciences, Cardiff University, Cardiff CF10 3AX, UK
*Correspondence: FoxKD@cardiff.ac.uk
http://dx.doi.org/10.1016/j.neuron.2015.09.025
This is an open access article under the CC BY license (http://creativecommons.org/licenses/by/4.0/).

SUMMARY

Layer 5 contains the major projection neurons of the neocortex and is composed of two major cell types: regular spiking (RS) cells, which have cortico-cortical projections, and intrinsic bursting cells (IB), which have subcortical projections. Little is known about the plasticity processes and specifically the molecular mechanisms by which these two cell classes develop and maintain their unique integrative properties. In this study, we find that RS and IB cells show fundementally different experience-dependent plasticity processes and integrate Hebbian and homeostatic components of plasticity differently. Both RS and IB cells showed TNFα-dependent homeostatic plasticity in response to sensory deprivation, but IB cells were capable of a much faster synaptic depression and homeostatic rebound than RS cells. Only IB cells showed input-specific potentiation that depended on CaMKII autophosphorylation. Our findings demonstrate that plasticity mechanisms are not uniform within the neocortex, even within a cortical layer, but are specialized within subcircuits.

INTRODUCTION

The cerebral cortex shows a remarkable capacity for functional plasticity (Feldman, 2009; Fox et al., 2000; Fox and Wong, 2005). Broadly, plasticity can take one of two forms: input-specific plasticity, which involves weakening of inactive inputs and strengthening (or weakening) of active inputs, and an input-agnostic form of plasticity, which involves both deprived and spared inputs and acts to maintain neuronal activity at some set point in a homeostatic fashion. Sensory cortex, where input to neurons can be conveniently manipulated by altering sensory experience, exhibits both forms of plasticity. In the barrel cortex, trimming the whiskers leads to rapid depression of the responsiveness of cortical neurons to deprived whiskers and a slower potentiation of responses to spared whiskers (Glazewski and Fox, 1996). Similarly in the visual cortex, monocular deprivation leads to rapid depression of cortical responses to closed eye input followed by slower potentiation of responses to both open and closed eye input (Kaneko et al., 2008). Input-specific and input-agnostic forms of functional plasticity map onto known synaptic plasticity mechanisms. Input-specific plasticity can be explained by Hebbian LTP and LTD and their spike timing-dependent forms (STDP), while input-agnostic plasticity can be explained by homeostatic synaptic scaling. Evidence for this view derives from studies where factors that are required for a particular form of plasticity are blocked or knocked out. For example, cortical LTP depends on auto-phosphorylation of CaMKII, and loss of this process in the CaMKII-t286a point mutant (Giese et al., 1998) blocks potentiation of spared whisker responses in layer 2/3 neurons as well as LTP (Hardingham et al., 2003) and disrupts ocular dominance plasticity in the visual cortex (Taha et al., 2002). Similarly, synaptic upscaling depends on TNFα, and knockout of *tnf*, or scavenging soluble TNFα, prevents homeostatic potentiation in visual cortex (Kaneko et al., 2008). While other forms of plasticity exist, such as changes in inhibition and changes in intrinsic membrane properties, LTP, LTD, and homeostatic mechanisms are demonstrably present in cortex and affect excitatory transmission within the cortex.

Theoretical considerations suggest that Hebbian plasticity requires homeostatic plasticity to maintain neuronal responses within a normal operating range (Turrigiano, 2008; Turrigiano et al., 1998). Hebbian plasticity, if left unchecked, would tend to drive synaptic weights to saturating maximum or minimum values, whereas homeostatic scaling would tend to normalize a cell's average response back toward a set point. Recent studies have questioned whether synaptic scaling can be involved in balancing Hebbian plasticity, however, because (a) synaptic scaling acts too slowly to stabilize the neuron following Hebbian plasticity and (b) blocking Hebbian plasticity does not lead to changes in synaptic weights (Chistiakova et al., 2015; Toyoizumi et al., 2014; Zenke et al., 2015). These considerations suggest a model in which Hebbian plasticity maintains neural activity levels at a cell specific set point over short timescales, while homeostatic synaptic scaling modulates the strength of neuronal inputs over a longer time envelope.

While these theoretical schemes are attractive, recent experimental evidence of cell type to cell type differences in plasticity profile, even within a cortical layer, suggest that a more nuanced description may be required. Specifically, Hebbian plasticity forms appear to be segregated between the two major

excitatory cortical cell types of layer 5, the regular spiking (RS) and the intrinsic bursting (IB) pyramidal cells of the cerebral cortex (Jacob et al., 2012). RS cells are distinguished by producing regularly timed trains of action potentials in response to somatic current injection; they tend to project cortically and have a distinct morphology comprising a slender apical dendrite with limited branches only near the cortical surface (Agmon and Connors, 1989, 1992; McCormick et al., 1985). In contrast, IB cells produce bursts of spikes to somatic current injection, project sub-cortically, and have complex apical dendrites that branch deeper in cortex, not just at the pial surface (Agmon and Connors, 1989, 1992; Connors and Gutnick, 1990; McCormick et al., 1985). RS cells tend to show strong synaptic depression in response to whisker deprivation, with little potentiation of spared whisker responses that affects only the short latency component. Conversely, IB cells show potentiation of responses to spared whiskers surrounding those that were trimmed and only limited depression to deprived inputs (Jacob et al., 2012). It is not at all clear how Hebbian plasticity alone could maintain these cells at a set activity level under these conditions without the existence of an opposite compensating plasticity mechanism.

We therefore sought to understand whether a homeostatic plasticity mechanism was present in RS and IB cells and whether it was possible to separate homeostatic from Hebbian components of plasticity. While RS and IB subtypes can be found in all cortical layers, we concentrated on layer 5 pyramidal cells. We used several methods to distinguish between homeostatic and Hebbian plasticity mechanisms. First, we studied the time course of whisker responses following whisker deprivation, reasoning that homeostatic plasticity should act to move the sensory response back toward the original levels, while Hebbian mechanisms should move spared and deprived whisker responses away from the original levels. Second, we measured whether changes were input specific and therefore Hebbian or were common to all inputs and therefore homeostatic. Third, we looked at the effect of deprivation on synaptic scaling to see whether changes were accounted for by alteration in excitatory synaptic weights and further, whether they scaled multiplicatively. Finally we sought to dissect the molecular basis of plasticity in both cell types by examining plasticity in CaMKII-t286a mutants that lack Hebbian potentiation and TNFα mutants that lack homeostatic upscaling.

Our findings revealed that both RS and IB cells exhibit homeostatic plasticity but that it operates at very different rates in the two cell types and that the propensity for Hebbian depression in RS cells is compensated for by homeostatic potentiation rather than Hebbian potentiation mechanisms. IB cells on the other hand showed Hebbian potentiation of spared whisker inputs in combination with homeostatic rebound of depressed inputs, which overall increased their synaptic drive in an uncompensated way, over the 10-day period we studied the process.

RESULTS

Experience-dependent plasticity in the barrel cortex is manifested as a change in the responses of neurons to stimulation of the whiskers within the receptive fields. We induced plasticity in barrel cortex by trimming the D-row whiskers (Figure 1A) for a period of 12 hr, 3 hr, or 10 days in C57/BL/6J mice. Recordings were made from the deprived columns, preferentially from D2, identified relative to the blood vessel pattern using intrinsic signal imaging (ISI) in order to target electrode penetrations (Figure 1A). We adopted the convention used by (Jacob et al., 2012) that the somatotopically related whisker for the recorded barrel is referred to as the principal whisker (PW) and its immediate in-row neighbors as T_1 and T_2, ranked according to strength of their spiking response. The whiskers in the flanking rows are designated as S_1–S_6. The trimmed whiskers in deprivation experiments are therefore PW, T_1, and T_2, and the undeprived whiskers are S_1 to S_6. The PW and the eight immediately surrounding whiskers were stimulated automatically with a pseudo-random sparse noise sequence (Figures 1C and 1D) to record a complete set of PSTHs and whisker evoked PSPs within 2 min (Figure 1E; see Experimental Procedures).

RS and IB subtypes of layer 5 cell were identified by their threshold response to somatic current injection (Figure 1B). While RS and IB cells were found throughout sublaminae 5A and 5B, there was a tendency for more IB cells to be recorded in 5B (Figure S1). However, in slice recordings from barrel cortex, it was clear that both subtypes were present in both sublaminae (Figure S1), and there were no differences in depth distribution for the RS and IB cells across the in vivo conditions described below (Figure S1).

Input specificity and time course of plasticity

To determine whether RS and IB cells showed Hebbian and homeostatic aspects of plasticity, we trimmed a single row of whiskers repeatedly over a 10-day period. This method creates an imbalance in the level of activity within the receptive field and in particular removes the strongest input (the PW) from the receptive field. After 3 days of row-deprivation, RS neurons showed maximum depression of both deprived and spared whisker responses (Figures 2A, 2B, S2A, and S2B; $F_{(3)} = 3.741$, $p = 0.0114$, average deprived and spared whisker responses, control versus 3 days: $q = 4.556$, ANOVA with Tukey's post hoc). After 10 days of deprivation, RS neurons showed a remarkable rebound potentiation back to control values despite the continued deprivation (Figure 2B, $q = 1.527$, $p > 0.05$ neither spared nor deprived significantly different from control, ANOVA with Tukey's post hoc). The rebound potentiation in RS cells and its time course are reminiscent of the homeostatic rebound potentiation reported previously for deprived eye responses in visual cortex (Kaneko et al., 2008; Ranson et al., 2012). Neither the original depression nor the rebound potentiation were input specific, affecting spared and deprived inputs alike (Figure 2A). RS cells therefore show two of the key characteristics of homeostatic plasticity.

Deprived inputs to IB neurons showed a similar behavior to that seen in RS neurons but with much faster kinetics (Figures 2C, 2D, S2D, and S2E). In IB neurons, deprived whisker responses were depressed after just 12 hr (Figures 2C and 2D; $F_{(3)} = 6.675$, $p < 0.0001$, $q = 3.935$) and recovered to control values by 3 days ($p > 0.05$, $q = 0.690$). Spared whisker responses followed approximately the same time course as deprived whiskers for RS cells, suggesting that a component of the surround whisker plasticity is not input specific and might instead be manifested as global modifications to the synaptic weightings of a

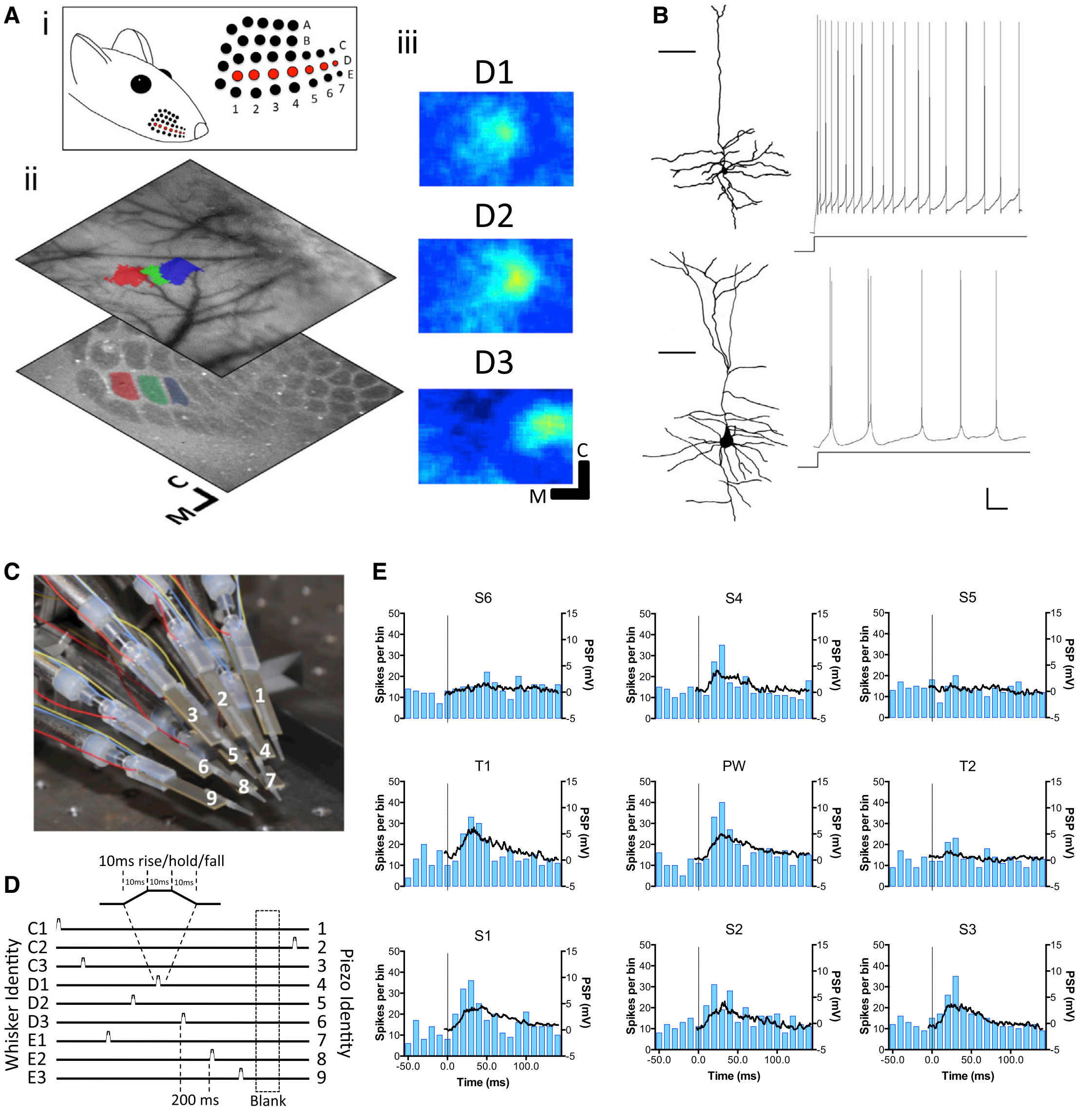

Figure 1. D-Row Whisker Deprivation Pattern, Whisker Stimulation, and RS/IB Cell Characteristics

(Ai) The D-row of whiskers are deprived from D1–7 (red circles) corresponding to the D-row of barrels.

(Aii) Electrode penetrations were targeted to the deprived barrels using ISI. Averaged responses from periodic stimulation are shown overlaid on the surface vasculature and example barrel field.

(Aiii) The magnitude map of the responses shown in the projection. The principal barrel was usually D2 (green area), and the remaining D-row whiskers (i.e., D1 [red], D3 [blue] areas) were designated T1 and T2, ranked in order of spike response strength. Similarly, the surround whiskers are designated S_1–S_6 based on their spike responses.

(B) Top: A camera lucida reconstruction of an RS cell with an example response to somatic current injection. The cell responds with a train of single spikes. Bottom: An example IB cell, which responds to current injection with bursts of high-frequency spikes punctuated by pauses in spike firing (scale bars: 150 μm for cells, 10 mV, and 200 ms for recordings). Cells recorded in vitro.

(C) The 3 × 3 piezoelectric whisker stimulator is centered on the PW to stimulate (usually) D2 and the eight surround whiskers automatically. The piezos are aligned to maintain the whiskers at their resting angle in the absence of stimulation.

(D) A sparse noise psuedorandom sequence is delivered in a group of ten (one for each piezo and a blank period). Each stimulus consists of a trapezoidal profile to reduce ringing (magnified trace at top).

(E) Example receptive field PSPs and PSTHs. Each graph is the average of responses over 50 repetitions of the stimulus sequence. Graphs are positioned in correspondence to the stimulated whisker (C1 at top left, E3 at bottom right) and labeled by classification based on supra-threshold response, deprivation status, and principal barrel. The vertical line indicates the time of the stimulus onset. (50 ms per histogram bar).

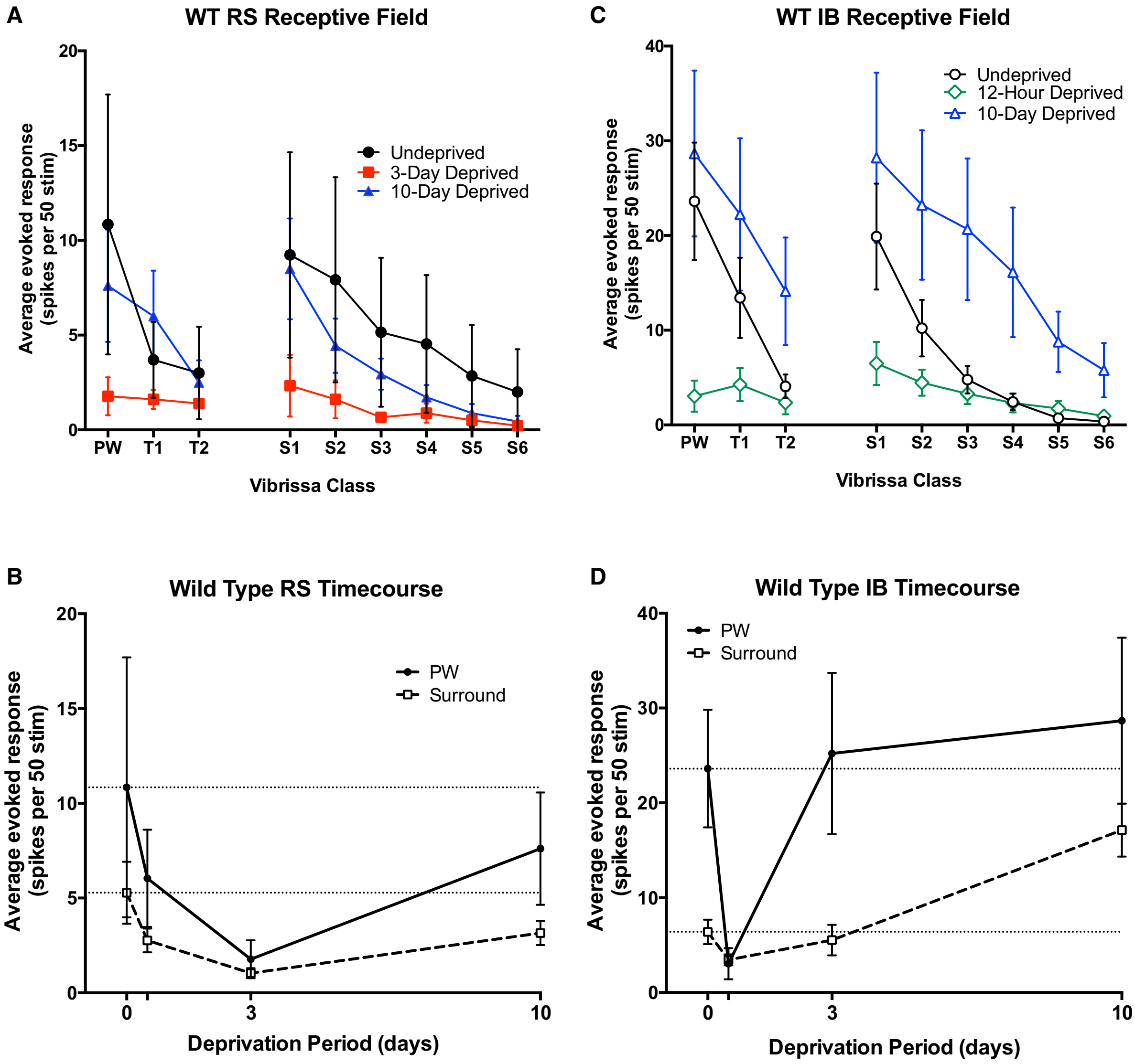

Figure 2. Receptive Field Characteristics and Time Course of Plasticity of RS and IB Cells in Normal and Deprived WTs

(A) Spike responses in RS cells. Receptive fields are shown for control (black), 3-day (red), and 10-day (blue) deprived conditions. The deprived whiskers are shown on the left (PW, T1, and T2) separated from the surround whiskers (S1–S6).

(B) RS cells, in both their deprived and spared inputs, display a slow depression of their spike responses between 0 and 3 days, with a partial recovery between 3 and 10 days.

(C) Receptive fields for IB cells. Control (black), 12-hr (green), and 10-day (blue) responses are shown. The 12-hr time point is plotted in place of the 3-day deprivation here, as this is the point of maximum depression in IB cells.

(D) In contrast to RS cells, IB cells depress quickly by 12 hr and then recover at 3 days deprivation. The surround inputs then display a strong potentiation between 3 and 10 days.

Error bars represent SEM.

cell (Figure 2). These findings suggest that IB cells also show homeostatic plasticity, but with a much faster time course than shown by the RS cells.

One aspect of the plasticity exhibited over this period was input specific. The spared whisker responses of the IB cells potentiated above baseline between 3 and 10 days. The spared whisker potentiation was significant (Figures 2C and 2D; Control versus 10 day, $p < 0.001$, $q = 6.773$, ANOVA with Tukey's post hoc) while the deprived whisker responses were not different from their control values (Control versus 10 day, $p > 0.05$, $q = 2.705$, ANOVA with Tukey's post hoc). Note that even though some of the deprived whisker responses look elevated (particularly T_2), they are not significantly different from control due to the relatively high variance in the distribution. Rather than a restorative form of potentiation, the potentiation between 3 and 10 days moved the responses away from their original

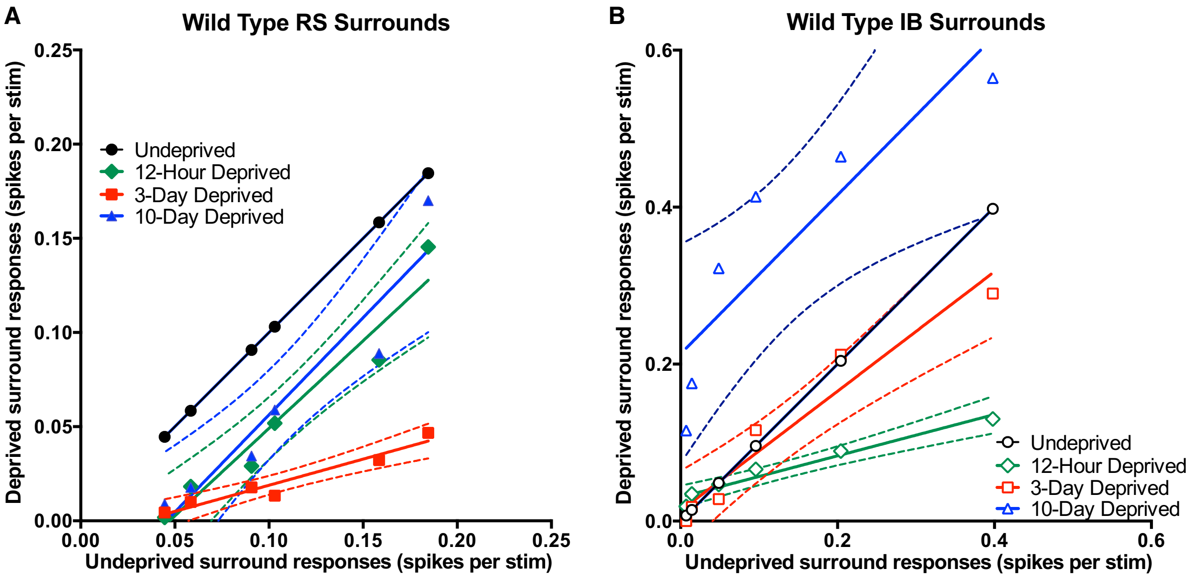

Figure 3. Surround Receptive Field Slope Plots for WT Mice

(A) Surround receptive field plots for WT RS cells. A change in slope is indicative of a multiplicative shift, whereas a change in intercept is more likely to be an additive or subtractive plasticity event. RS surrounds show a downward parallel shift from control to 12 hr, followed by a slope change downward to 3 days. This slope change is then reversed between 3 and 10 days, with the 10-day response plot being identical to the 12-hr one.

(B) IB surround plots highlight a possible mechanistic difference between RS and IB cell plasticity. The depression between 0 and 12 hr is a slope change, reversed by 3 days. The potentiation between 3 and 10 days is best represented by a parallel shift.

Dashed lines represent 95% confidence limits.

values. These features suggest that IB cells show an additional Hebbian potentiation component to their plasticity.

Surround Receptive Field Transforms

To investigate further the nature of the plasticity, we analyzed the surround receptive field responses by response magnitude. Different whiskers in the receptive field naturally drive the neuron with different intensity, generating a range of response amplitudes. If plasticity scales the responses by a common factor, as with homeostatic scaling, then small responses should be scaled by the same factor as large responses. If the transform is other than proportional it could be indicative of another type of plasticity mechanism such as LTP or LTD. We therefore asked if the plasticity between the deprivation time points could be described by a multiplicative transform.

For the RS cells, we plotted the average spared receptive field (surround whiskers one to six, $S_{1–6}$) recorded after 12 hr of deprivation against their respective average control responses, and obtained a linear relationship between the two with an almost identical slope (Figure 3A, 12 hr deprivation: slope = 0.929 ± 0.118, R^2 = 0.94, $F_{(1)}$ = 0.35, p = 0.57, ANOVA), suggesting that a multiplicative process could not describe the initial change at 12 hr. Each whisker response had shifted to a lower value by a similar quantity and therefore the y intercept for the lines were significantly different ($F_{(1)}$ = 80.74, $p < 0.0001$), indicating a subtractive transformation (possibly indicative of LTD). At 3 days deprivation, the $S_{1–6}$ responses decreased further and this time did show a decrease in slope (3 days deprivation: for linear fit R^2 = 0.94, $F_{(1)}$ = 28.01, p = 0.0007), indicative of a multiplicative transformation. This was then reversed between 3 and 10 days without restoring the subtractive initial depression (10 day deprivation: for linear fit, R^2 = 0.90, slope comparison $F_{(1)}$ = 0.249, p = 0.63, 10 day slope not significant versus 12 hr. Intercept $F_{(1)}$ = 0.626, p = 0.45 versus 12 hr), which implied that the rebound potentiation was proportional to the depressed values and that a multiplicative transformation had once again occurred (Figure 3A). Such proportional changes might be observed if global multiplicative scaling were to underlie the homeostatic rebound (Ranson et al., 2012; Stellwagen and Malenka, 2006; Turrigiano et al., 1998).

The IB cells displayed both a biphasic and an input-specific series of changes. The $S_{1–6}$ responses were significantly depressed after 12 hr in a manner that could be described by a reducing multiplicative transform (Figure 3B, linear fit, R^2 = 0.9563, slope comparison $F_{(1)}$ = 687.3, $p < 0.0001$) and recovered toward control levels after 3 days (Figure 3B, linear fit, R^2 = 0.9383, $F_{(2)}$ = 0.7391, p = 0.498). The $S_{1–6}$ responses then potentiated above baseline after 10 days (Figures 2C, 2D, and 3B) without a further change in slope (Figure 3B, 10 days deprivation: linear fit, R^2 = 0.7812, slope comparison $F_{(2)}$ = 0.7391, p = 0.4981).

The IB cell potentiation between 3 and 10 days was more closely fit by a vertical shift as reflected in the significant change from the undeprived case in the y intercept but not the slope (Figures 3B and S3 10 days deprivation: intercept = 0.2130 ± 0.05, $F_{(2)}$ = 33.8214, $p < 0.0001$). These observations further emphasize that two different modes of plasticity are present in the RS and IB cells that operate at different time points in the sequence

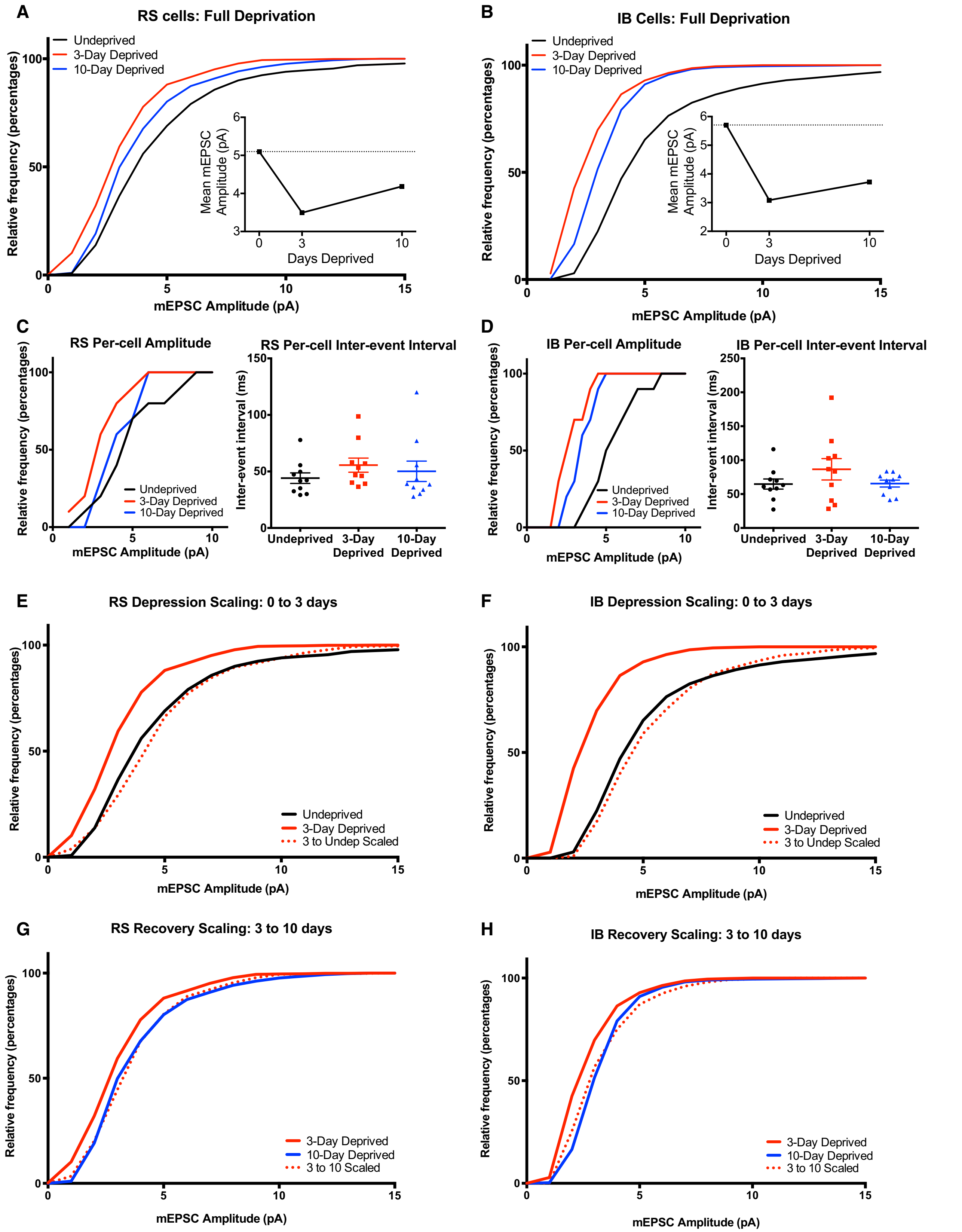

(legend on next page)

of depression and homeostatic rebound for the RS cells and depression and potentiation for the IB cells. The S_{1-6} vector for the RS cells shows a downshift followed by multiplicative decrease and multiplicative recovery, while the IB cells show a multiplicative decrease, followed by a multiplicative recovery and non-multiplicative potentiation. These data suggest that we should expect to find two different types of plasticity mechanism operating during sensory deprivation in layer 5 cells.

Homeostatic Synaptic Scaling in the Barrel Cortex In Vitro

One component of the changes in sensory responses in vivo is characteristic of homeostatic plasticity. Theoretically, several different mechanisms could underlie such homeostatic changes including changes in inhibition, changes in intrinsic membrane properties, or synaptic scaling of excitatory responses. We tested the last possibility, and as an initial test for the existence of synaptic scaling in RS and IB cells, we performed complete unilateral whisker trimming for 3 or 10 days duration and then prepared slices of contralateral barrel cortex in order to record mEPSCs. The complete deprivation leaves no input-driven changes, and instead highlights the intrinsic, input-independent global response of the recorded cells. In RS and IB cells, we found that mEPSC amplitudes were significantly depressed after 3 days of complete whisker trimming (Figure 4, comparing mEPSC amplitude day 3 and control, RS: $p < 0.001$, $D = 0.556$, IB: $p < 0.01$, $D = 0.625$, KS test). In common with the recovery of responses in vivo, mEPSC amplitudes recovered toward control values after day 10 complete whisker trimming, indicative of both cell types exhibiting homeostatic synaptic scaling (Figures 4A–4D).

RS cells did not show multiplicative downscaling as the control, and 3-day distributions could not be scaled to each other, (Figure 4E; 3 day scaled is different from control, Scale factor = 0.668, $D = 0.3889$, $p < 0.01$, KS test), but they did show multiplicative upscaling from the depressed state between 3 and 10 days (10 day is not different from 3 day scaled, Scale factor = 1.145, $D = 0.083$, $p = 0.98$, KS test). IB cells showed both multiplicative downscaling between 0 and 3 days complete whisker trimming and rebound upscaling between 3 and 10 days (Figures 4F and 4H; 3 day scaled is not different from control, scale factor = 0.540, $D = 0.2581$, $p = 0.253$; 10 day is not different from 3 day scaled, scale factor = 1.206, $D = 0.095$, $p = 0.98$, KS test). Complete whisker trimming did not cause a change in inter-event interval in either cell type at any time point (Figures 4C and 4D, RS cells KW = 3.254, $p = 0.1965$, IB cells KW = 1.683, $p = 0.4312$, Kruskal-Wallis test). This experiment shows that multiplicative homeostatic scaling occurs in layer 5 of the barrel cortex. Upscaling from the depressed state can be described by a simple multiplicative gain change in synaptic weights for RS and IB cells, which is consistent with the homeostatic process we observe in vivo. However, the non-multiplicative downscaling only seen in the RS cells is consistent with the non-multiplicative downward shift seen between 0 and 12 hr in the RS cell spike responses (Figure 3A).

mEPSC Amplitudes in D-Row-Deprived Wild-Type Cells

To study the changes in mEPSCs under similar conditions to those used to induce plasticity in the in vivo experiments, we repeated the study but this time in mice deprived of a single row of whiskers. After 3 or 10 days deprivation, cortical slices were prepared and recordings made specifically from layer 5 cells in deprived (D-row) columns. RS cells showed depression of mEPSC amplitudes after 3 days (Figure 5A, RS = 5.10 ± 0.11 pA control versus 3.15 ± 0.04 pA 3 day, $D = 0.694$, $p < 0.001$, KS test) and significant recovery to a level near control after 10 days deprivation (4.56 ± 0.10 pA, $D = 0.361$, $p = 0.018$ versus 3 day, KS test). The mEPSC amplitude behavior therefore completely recapitulated the changes in spike firing seen in the studies in vivo (Figure S4). For IB cells, the mEPSC amplitude distribution did not show depression after 3 days of row deprivation (Figure 5B, IB = 5.17 ± 0.10 pA control versus 3 day 5.87 ± 0.08 pA, $D = 0.35$, $p = 0.17$, KS) but did show a strong increase in mEPSC amplitudes after 10 days (7.48 ± 0.16 pA, $D = 0.500$, $p = 0.013$, KS), again mimicking the spike firing changes in vivo. Deprivation did not cause a change in inter-event interval in either cell type at any time point (Figures 5C and 5D; RS cells KW = 5.086, $p = 0.08$, IB cells KW = 5.24, $p = 0.07$).

We looked at whether the mEPSC amplitudes scaled in a multiplicative manner between any of the time points (Figures 5E–5H). We found that row deprivation resulted in changes in amplitude distribution that did not scale multiplicatively, neither the depression between control and 3 days of row deprivation (RS, $D = 0.389$, $p = 0.008$, KS) nor the potentiation between 3

Figure 4. mEPSC Activity in WT Mice Subject to Complete Unilateral Whisker Deprivation Suggests that Synaptic Scaling Is Present in Both RS and IB Cells

(A) Cumulative distribution functions of miniature events in RS cells. The progression of the amplitude of the RS mEPSCs reflects the spike responses seen in vivo with row deprivation; an initial depression at 3 days is followed by a partial recovery by 10 days. The time course of the mean mEPSC amplitude can be seen in the inset.

(B) IB cells do not parallel the row-deprived in vivo spike phenotype. At 3 days, there is a significant depression of mEPSC amplitude, which is slightly recovered by 10 days.

(C) Per-cell amplitudes of RS cells (left) reflect the phenotype displayed by the pooled data. No difference was observed in the inter-event intervals of the mEPSCs (right).

(D) Again, in IB cells, per-cell amplitude data (left) strongly reflects the pooled data, with no effect of deprivation on inter-event interval (right).

(E) Depression in RS cells cannot be explained solely by synaptic scaling. The 3-day deprived CDF cannot be multiplied to be identical to the control data.

(F) In contrast, the depression observed in IB cells in this preparation can be attributed to scaling. Multiplying the 3 day data by 1.850 leads to a CDF that is not significantly different from the control distribution.

(G and H) Both RS and IB cells scale their responses between 3 and 10 days. Multiplying RS 3 day data by 1.145 and the IB 3 day data by 1.206 leads to CDFs that match their respective 10-day deprived distributions.

In (C) and (D), the long horizontal lines indicate means and the short horizontal lines SEM.

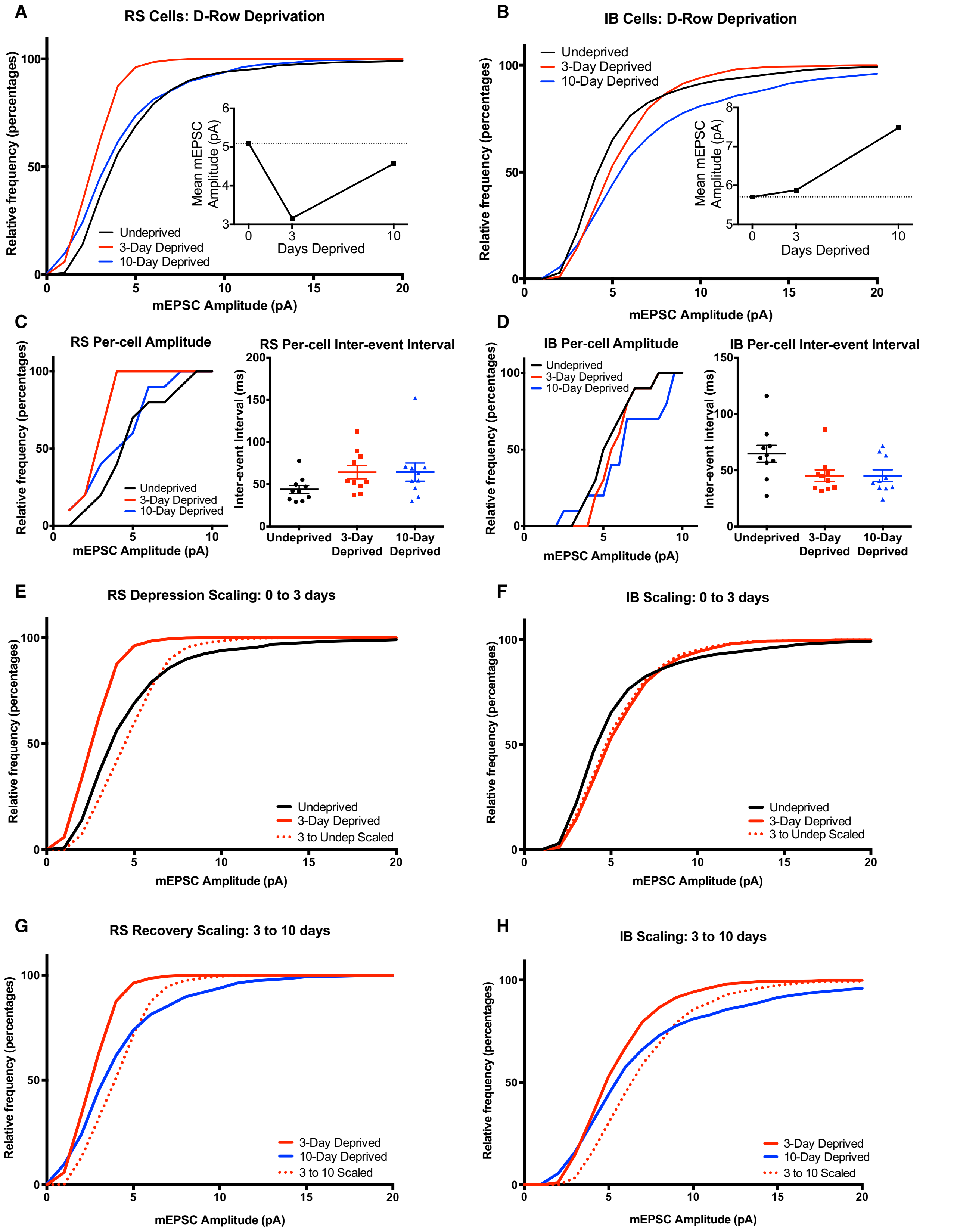

(legend on next page)

and 10 days (RS, D = 0.15, $p < 0.01$, IB D = 0.16, $p < 0.01$, KS). These results show that row deprivation leads to more complex changes than removing all the whiskers evenly. This is perhaps inevitable, as all inputs are sampled in a mEPSP recording including inputs driven by spared and deprived whiskers, and we know from the recordings in vivo that only some of the plasticity transforms are common across inputs. Nevertheless, the time course of plasticity in the average mEPSC amplitudes and their direction of change at each time point mimicked the changes in sensory-evoked spike firing seen in vivo in an IB- and RS-cell-specific manner (Figure S4), implying that changes in excitatory responses were sufficient to explain the changes in receptive fields seen in vivo.

Plasticity in tnf Knockout Mice

Homeostatic plasticity occurs in visual cortex during the critical period (Kaneko et al., 2008) and is characterized by a rebound of the response to a deprived input (closed eye) despite continued deprivation, analogous to the rebound of the response to the trimmed whisker input seen here. In the visual cortex, this form of homeostatic plasticity is due to synaptic scaling and depends on TNFα both in vitro (Stellwagen and Malenka, 2006) and in vivo (Kaneko et al., 2008). The rebounds seen in both RS and IB cells in WTs could well be explained by a homeostatic response to the initial depression. To test whether RS and IB cells make use of TNFα to generate homeostatic plasticity, we repeated the D-row deprivation experiments in *tnf* knockout mice. In RS cells, while the depression occurred normally after 3 days of deprivation, the response did not recover after 10 days (Figures 6A–6C). Consequently, the slope of the $S_{1–6}$ function was depressed and approximately the same at 3 days as at 10 days of deprivation (Figures 6B; 3 days deprivation: slope comparison, $F_{(1)} = 3.77$, $p = 0.087$). An ANOVA for duration of deprivation and genotype showed an effect of deprivation and an interaction between genotype and deprivation (Deprivation: $F_{(2)} = 22.69$, $p < 0.0001$, Interaction: $F_{(2)} = 5.743$, $p = 0.0034$). The interaction term arises from the difference in response at 10 days deprivation in the *tnf* KOs versus the WTs ($t_{(52)} = 3.144$, $p = 0.0028$, t test). These findings demonstrate that TNFα is necessary for homeostatic rebound potentiation in RS cells.

Both spared and deprived inputs in IB cells showed depression at 3 days, unlike WTs (Spared control versus 3 days: average whisker responses $F_{(2)} = 5.435$, $p = 0.005$, $q = 4.175$, ANOVA with Tukey's post hoc), suggesting that recovery from depression at 12 hr was at least partly TNFα dependent (Figures 6D–6F). However, the depressed responses were able to recover to baseline values after 10 days of deprivation in the absence of TNFα (Figure 6F, spared control versus 10 days: $p > 0.05$, $q = 0.1850$), implying a second synaptic mechanism is involved in the IB cells that is not active in the RS cells.

Plasticity in CaMKII-t286a mice

The results in vitro suggest that, in RS cells, most of the changes seen in response to deprivation are cell-intrinsic. In vivo, RS cell recovery between 3 and 10 days is dependent on TNFα. In IB cells, the rebound-potentiation of deprived inputs between 12 hr and 3 days can also largely be explained by a TNFα-dependent form of plasticity. However, spared whisker responses potentiate even in the absence of TNFα (Figures 6E and 6F), and spared whisker potentiation in the wild-type (WT) mice cannot be described as a uniform scaling of all inputs (Figure 3B). These findings— along with the difference observed between deprivation protocols in vitro—suggest a second mechanism is involved in potentiation in the IB cells. In layer 2/3 of the barrel cortex, experience-dependent potentiation and LTP are both dependent on autophosphorylation of CaMKII as is LTP in the hippocampus, and visual cortex (Giese et al., 1998; Glazewski et al., 2000; Hardingham et al., 2003; Kirkwood et al., 1997; Taha et al., 2002) and open eye potentiation in the adult mouse visual cortex (Ranson et al., 2012). This mechanism is a strong candidate for driving the potentiation of spared inputs in IB cells. We therefore tested the effect of D-row deprivation on RS plasticity and specifically on IB cell potentiation in CaMKII-t286a point mutants that lack autophosphorylation of CaMKII and have an LTP deficit.

The RS cells did show a rebound potentiation at 10 days from depression at 3 days in the CaMKII-t286a mice, suggesting that it does not depend on an LTP like process in this cell type (Figures 7A–7C). However, the recovery was most apparent in response to strong inputs (PW, S_1, and S_2) and absent in the minor surround whisker responses $S_{3–6}$, which may require an LTP like potentiation mechanism to recover to baseline.

In IB cells, in genetically altered animals, again in contrast to WTs, depression occurred at 3 days deprivation, suggesting a rapid ongoing plasticity in IB cells to maintain baseline responses that not only requires TNFα (Figure 6) but also CaMKII (Figures 7D–7F). Second, the lack of CaMKII autophosphorylation prevented the potentiation of the $S_{1–6}$ responses (Figure 7E). An ANOVA for WT and CaMKII-t286a animals showed an effect of deprivation and an interaction between deprivation and genotype (Deprivation: $F_{(2)} = 9.426$, $p = 0.0001$, Interaction: $F_{(2)} = 6.550$, $p = 0.0016$). Post hoc tests showed that the interaction term arose due to the lack of potentiation at the 10 day time point in the CaMKII mutants (17.13 ± 2.79 versus 6.01 ± 1.42 spikes, $q = 6.666$, Tukey's post hoc test).

Figure 5. D-Row Deprivation Creates Strong Parallels between mEPSC and In Vivo Spike Data

(A) RS cell mEPSCs show a marked depression at 3 days and a strong recovery by 10 days, reflecting both the in vivo data and that seen in full deprivation slice preparations.

(B) In contrast to the progression of mEPSC amplitude in the complete whisker trimming experiment, IB cells show little change in amplitude at 3 days and a large potentiation by 10 days deprivation (time course in inset). This is reminiscent of the progression seen in the spikes recorded in vivo in D-row deprived WT mice.

(C) Per-cell amplitudes of RS mEPSCs (left) are similar to the grouped CDFs, with no significant change in the per-cell IEI with deprivation (right).

(D) Similarly, in IB cells, the per-cell distribution (left) is similar to the pooled data, and there is no significant change in IEI (right).

(E–H) In both IB and RS cells, none of the amplitude changes observed in D-row-deprived mEPSC recordings can be explained simply through scaling. It is not possible to multiply any of the CDFs to resemble any others.

In (C) and (D), the long horizontal lines indicate means and the short horizontal lines SEM.

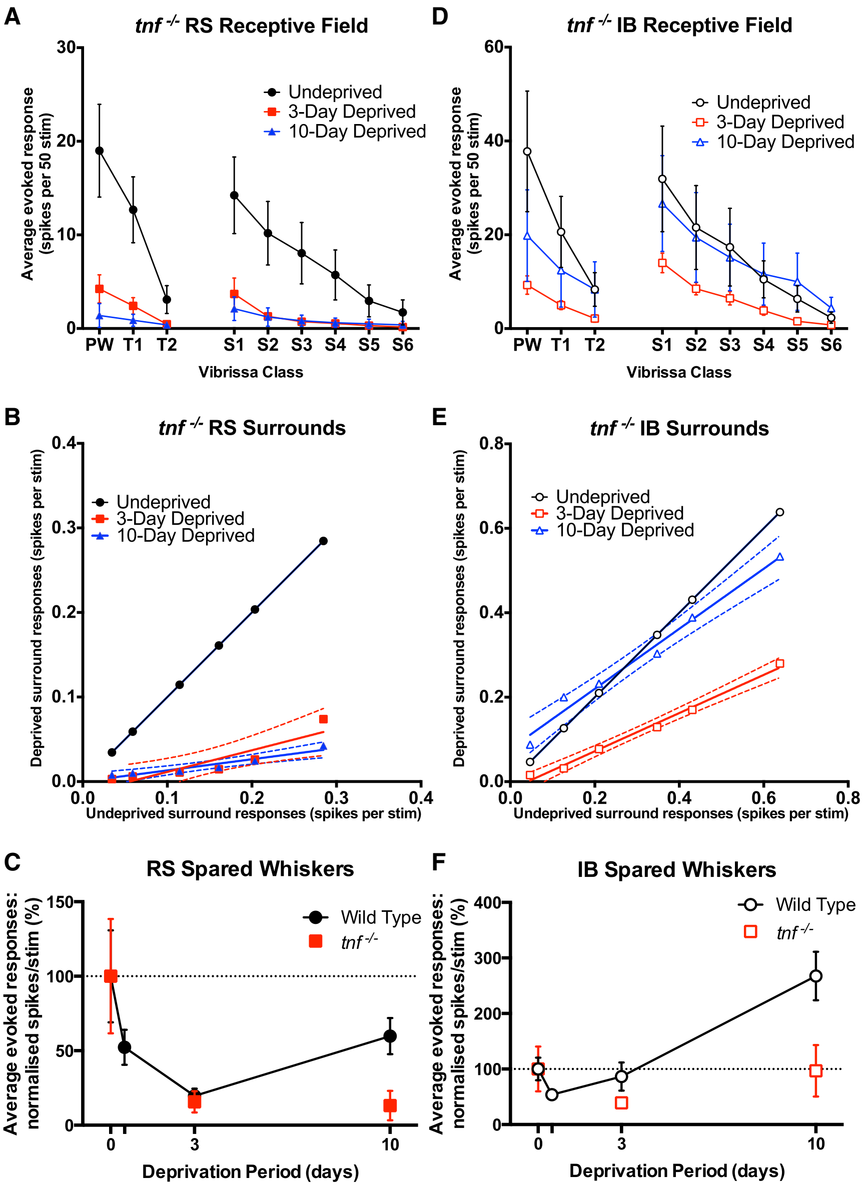

Figure 6. Plasticity in *tnf* Knockout Mice

(A) Whisker responses of RS cells recorded in *tnf* knockout mice fail to recover from spike rate depression at 10 days, and responses are still significantly depressed compared to control responses.

(B) In RS cells, the slope of the S1–6 response vector decreases after 3 days deprivation with no recovery in slope after 10 days deprivation.

(C) Normalized WT versus *tnf* knockout mice responses in spared inputs of RS cells. A strong correlation can be seen at 0 and 3 days, with a noticeable divergence at 10 days due to the lack of recovery in the mutant animals.

(D) Whisker responses in IB cells recorded in *tnf* knockouts show depressed spike rates after 3 days deprivation, which do recover to baseline after 10 days but do not potentiate in contrast to cells in WT animals.

(E) The mechanism of recovery of depression in IB cells lacking TNFα differs from that seen in RS cells in WT mice. After 3 days, the slope of the S1–6 vector is reduced. After 10 days, the surround responses recover toward baseline, but not by an exclusively multiplicative mechanism.

(F) Normalized WT versus *tnf* knockout mice responses in spared inputs of IB cells. In contrast to WT cells, the *tnf* knockout responses are depressed at 3 days, then stage a recovery back to baseline at 10 days. By 10 days deprivation, WT IB cells have strongly potentiated in their responses to surround inputs.

Error bars represent SEM in (A), (C), (D), and (F). Dashed lines represent 95% confidence limits in (B) and (E).

In conclusion, IB cells show a high level of dependence on CaMKII autophosphorylation for potentiation of responses (Figures 7D–7F), but this operates in conjunction with TNFα-dependent mechanisms, which might explain the faster rate of recovery from depression compared to RS cells. The RS and IB cells show different functional plasticity in the barrel cortex, and these differences depend in turn on different molecular mechanisms operating in the cells: almost entirely TNFα-dependent homeostatic plasticity in the RS cells and an additional more classical CaMKII-dependent component for the spared whiskers in the IB cells.

DISCUSSION

Previous studies have shown that synaptic weights can be modified by one of two general classes of mechanism, on the one hand a classical Hebbian form of plasticity characterized by LTD/LTP-like processes and on the other homeostatic potentiation characterized by a synaptic scaling process. In this study, we have sought to distinguish between the two subtypes of potentiation by a variety of methods. We found evidence for both homeostatic and Hebbian forms of plasticity in RS and IB cells in cortical layer 5. Homeostatic plasticity was common to both cell types but exhibited much faster kinetics in IB cells. Hebbian components differed between the two cell types. RS cells showed a form of spike response depression that could not be explained by a multiplicative transform. Similarly, the control mEPSC amplitude distribution could not be scaled onto depressed values, and the initial depression of surround whisker responses at 12 hr was best described by a uniform decrease in sensory response values (LTD-like) and not by a slope change. This form of depression was not seen in IB cells, which only showed a multiplicative decrease and rapid recovery in sensory responses. In the IB cells, the Hebbian component of plasticity comprised potentiation of spared whisker input, which was CaMKII dependent and therefore related to LTP. CaMKII-dependent potentiation was not present in the RS cells. These findings challenge the notion that LTP and LTD might be in equilibrium in

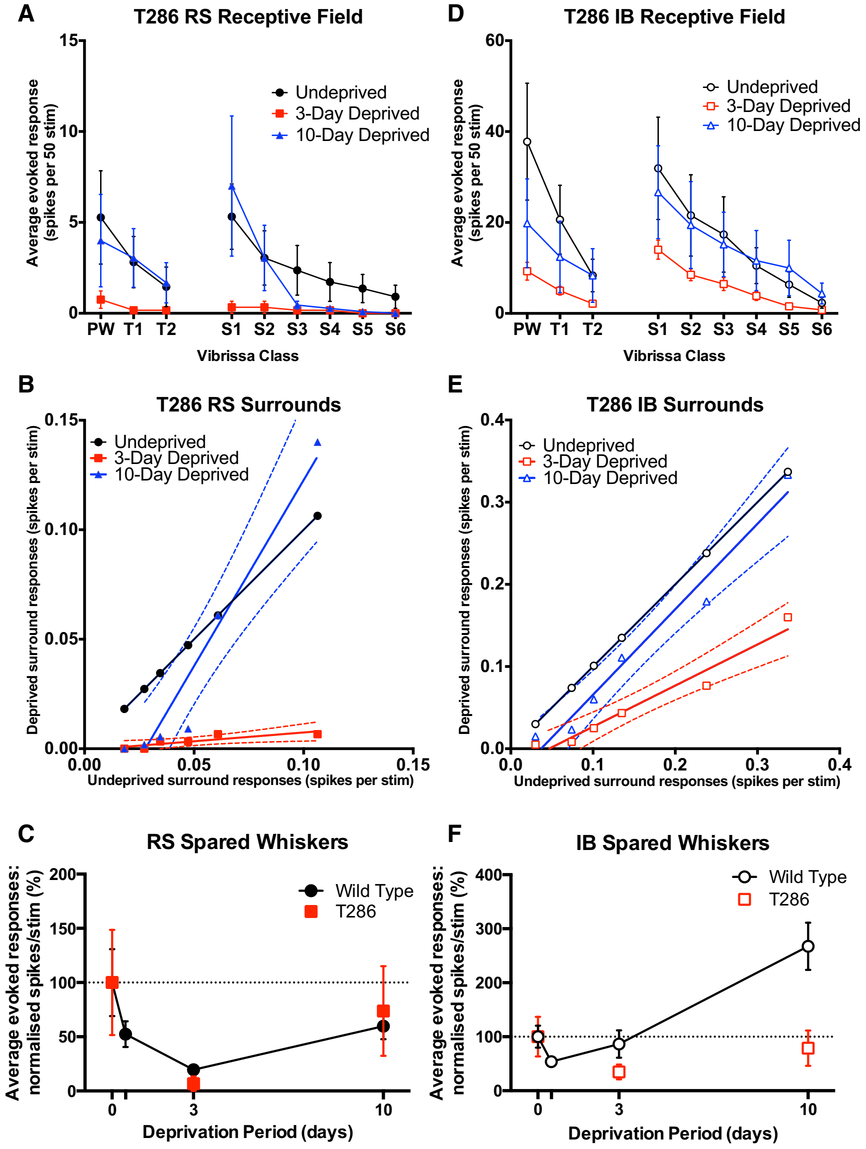

Figure 7. RS and IB Cells Respond Differentially in CamKII-t286a Mice

(A) RS cells display a depression and recovery phenotype similar to cells in WTs. Much of the recovery is driven by the strongest two surround whiskers in this case.

(B) RS surround plots show a very similar response at 3 days in CamKII mutants as in WT mice. The S1-S2 driven recovery at 10 days is also evident.

(C) Normalized spike rates for WT and CamKII mutant mice. The progression of RS responses in CamKII mutants is very similar to that of WT mice.

(D) In IB cells, mutation of CamKII leads to a plasticity phenotype similar to that of the *tnf* knckout mice. A depression at 3 days is followed by a partial recovery at 10 days.

(E) The IB surrounds depress at 3 days, with the recovery driven by a change in slope between 3 and 10 days. This is consistent with the expected reliance on multiplicative mechanisms given the hampered potential for LTP-like plasticity in these animals.

(F) In contrast, IB cells show a divergence between WT and CamKII-t286a mice at 3 days and especially at 10 days.

Error bars represent SEM in (A), (C), (D), and (F). Dashed lines represent 95% confidence limits in (B) and (E).

order to maintain homeostasis of cellular responses because LTD and LTP processes appear separated by cell type. Instead, we have observed that homeostatic regulation of an LTD-like depression of response is returned to control values by a TNF-α-dependent upscaling process in RS cells.

Hebbian and Homeostatic Plasticity Components In Vivo

We have primarily used five criteria derived from the literature to make the distinction between Hebbian and homeostatic potentiation. For homeostatic plasticity, these are that (1) the changes will be input agnostic (i.e., deprived and spared inputs will move in the same direction); (2) if perturbed, the input will move in a direction to restore the original values; (3) the factor will asymptote to baseline values; and (4) homeostatic plasticity will be TNFα dependent and (5) not CaMKII-autophosphorylation dependent. In addition, we have introduced a new indicator in this study, which is that (6) the surround receptive field vector shows a change in slope. As shown in Figure S3, the $S_{1–6}$ slope changes show a remarkable similarity to the time course of the putative homeostatic plasticity, but not the potentiation beyond baseline by the IB cells. Potentially this method is could be generalized to other systems where the input can be varied systematically (for example, orientation in the visual system or pitch tuning in the auditory system).

Plasticity observed between 3 and 10 days in the RS cells in vivo satisfies all six of the criteria for homeostatic potentiation. There was a strong, input-independent rebound toward the original response level without further potentiation. This was critically dependent on TNFα function and independent of CaMKII autophosphorylation. This suggests that TNFα-dependent homeostatic potentiation is sufficient to explain the potentiation. Additionally, the slope of the surround receptive field response vector changed in close correspondence with the overall spike responses of the cells (Figure S3).

Plasticity in the IB cells partly fit the criteria for homeostatic plasticity at the earlier time points. Rebound potentiation between 12 hr and 3 days was not trimmed-whisker specific, reacted back toward the original values, was TNFα dependent, and was associated with a slope change in the $S_{1–6}$ vector. In our previous study (Jacob et al., 2012), it was puzzling that IB cells might not exhibit synaptic depression after input deprivation, but the present results clarify this issue by demonstrating that IB cells are capable of depression (Figure 2D, 12 hr time

point) but that they react very quickly to compensate for it. Homeostatic plasticity in IB cells is therefore much faster in IB than in RS cells, and this may be due to the higher baseline firing rates shown by IB neurons that create a greater error signal following deprivation and therefore a greater drive for homeostatic potentiation. In this respect, it is interesting that the RS and IB cells each maintain a different set point and return to it following deprivation. For example, the average PW response of an IB cell is more than twice that of an RS cell (Figures 2A and 2C). These findings suggest that cells contributing to independent circuits within the cortex are able to regulate their set points quite independent of one another. Given that RS and IB cells are in close proximity and even sit within the same dendritic mini-columns (Krieger et al., 2007), this suggests that homeostatic factors such as TNFα are able to operate locally in a cell-specific manner.

Relationship between Sensory Response Plasticity and mEPSCs

Remarkably, the changes in spike firing in the two types of cell mirrored the changes seen in mEPSCs, implying that the depression and potentiation observed in spiking in vivo can in large part be accounted for by decreases or increases, respectively, in the weights of excitatory synapses. The changes in whisker responses can therefore be explained by intrinsic changes in the layer 5 neurons' excitatory synapses without the need to invoke circuit changes as causal agents. This conclusion is consistent with what is known about circuit changes in the rest of the cortex during row-deprivation. Layer 2/3 neurons form a major input to layer 5, but since they do not show depression after just 3 days row deprivation (Jacob et al., 2012), they cannot contribute to the depression seen in layer 5 RS cells at this time point. Layer 2/3 cells do show considerable depression after 10 days deprivation, which suggests that the homeostatic recovery shown in layer 5 cells occurs despite this reduction in input (Jacob et al., 2012). Regarding potentiation, layer 2/3 cells do not show potentiation of spared whisker input with row-deprivation (Drew and Feldman, 2009; Jacob et al., 2012), and therefore the layer 5 IB cells' potentiation cannot passively reflect potentiation of spared input from this source. However, circuit mapping in cortical slices using caged glutamate shows that synaptic input onto layer 5 neurons is strengthened from intracortical sources after 10 days of deprivation, including that from layer 2/3 neurons in surrounding barrels (Jacob et al., 2012), which implies that surround whisker potentiation is due to synaptic potentiation of inputs onto layer 5 cells.

Multiplicative scaling is a property that often accompanies homeostatic plasticity (Turrigiano et al., 1998). For homeostatic plasticity to maintain the relative weights of the synapses undergoing scaling and therefore any information they might encode, the synaptic weights should all scale by a common factor (Turrigiano et al., 1998). The RS cells did not show multiplicative scaling of the mEPSCs in the row-deprivation condition. Nevertheless, the mEPSC distribution moved in close correspondence with the changes seen in spike firing (Figure S4), showing that excitatory scaling of heterogenous synaptic inputs, albeit not multiplicative, could underlie the changes in spike firing.

Cellular Mechanisms Underlying Homeostatic and Hebbian Plasticity

We used the natural variation in response level produced by different whiskers within the receptive field to determine whether whisker deprivation scaled the receptive field responses proportionately. In the case of the homeostatic plasticity changes in the RS cells, the changes we observed could best be accounted for by changes in the gain of the responses; in other words, all the inputs scaled uniformly. However, it was not possible to explain the data for the IB cells in the same way. Instead, the IB cells responses tended to increase by the same amount independent of their response level, and were best fit by a parallel shift in the response vector (Figure 3B). This finding is in accordance with modeling studies that have shown how interactions between Hebbian plasticity (LTP and LTD) and homeostatic plasticity processes can be modeled by additive Hebbian and multiplicative homeostatic plasticity (Toyoizumi et al., 2014). Indeed, the cellular mechanisms underlying these two types of plasticity are likely to lead naturally to the two different computational functions. There is evidence that LTP can lead to increases in the number of spines or the input-specific gain of individual spines, both functions that add excitatory drive to the cell (Engert and Bonhoeffer, 1999; Matsuzaki et al., 2004). Similarly, IB cells show addition of new persistent spines following whisker deprivation, a process that is absent in CaMKII-t286a mice (Holtmaat et al., 2006; Wilbrecht et al., 2010) that lack LTP (Giese et al., 1998; Hardingham et al., 2003). Conversely, the TNF-α-dependent homeostatic plasticity mechanism acts via synaptic scaling, which is a multiplicative process leading to a proportional increase or decrease of synaptic weights (Kaneko et al., 2008; Ranson et al., 2012; Turrigiano et al., 1998). Current evidence suggests that this type of homeostatic plasticity may act by changing the dimensions of pre-existing spines rather than adding new ones (Keck et al., 2013).

Age and Layer Dependency of Homeostatic Plasticity

Our findings demonstrate that homeostatic plasticity occurs in the cortex of adult mice. This result contrasts with the conclusions of studies in visual cortex that showing that ocular dominance plasticity is only TNFα dependent in juvenile animals during the critical period and not in adulthood and therefore that homeostatic potentiation does not occur in adult animals (Ranson et al., 2012). The resolution of this discrepancy may be that different layers exhibit different modes of plasticity in adulthood. Layer 5 neurons show synaptic scaling in response to eye enucleation in adult animals (P100–P120) (Keck et al., 2013), while cells in layers 2/3 and layer 4 show early critical periods for synaptic scaling (Desai et al., 2002). Since the intrinsic imaging signal used to measure ocular dominance plasticity is almost entirely derived from layers 2/3 and 4 cells, the layer 5 TNFα-dependent component would not have been detected in adults using this method (Ranson et al., 2012).

CONCLUSIONS

In conclusion, these studies show molecular mediators of two different types of dynamic response to whisker deprivation in the mouse barrel cortex. The more classical potentiation

mechanism seen here in the IB cells, and previously in the L2/3 cells of barrel cortex (Glazewski et al., 2000), is dependent on CaMKII autophosphorylation. The homeostatic potentiation mechanism present in the RS cells is TNFα dependent and does not require CaMKII autophosphorylation. These studies raise questions about the differing functional requirements of the cortico-cortically projecting RS cells versus the sub-cortically projecting IB cells that require such different plasticity responses and that in turn require differing molecular mechanisms with which to underpin them.

EXPERIMENTAL PROCEDURES

A brief description of the methodology is given below, but full methods are available in the Supplemental Information.

Subjects and Whisker Deprivation

All procedures were approved under the UK Animals (Scientific Procedures) Act 1986. A total of 120 mice were used in the study. All mice were either WT Jackson C57Bl/6J (Charles River) or backcrossed into the Jackson C57Bl/6J background. Whisker deprivation was achieved by trimming the D-row under light isoflurane anesthesia every 24–48 hr. Trimmed whiskers were replaced by their contralateral equivalents before recording, reattached with cyanoacrylate glue.

In Vivo Recording

WT, *tnf*, and CamKII-t286a mice were anaesthetized with urethane (1.0 g/kg) and a trace amount of acepromazine. The parietal cranium was exposed, and the D-row location relative to the cranial vasculature was obtained using periodic-stimulus ISI with 700 nm light. A small craniotomy was made over the likely location of the D2 barrel and the dura resected with a hypodermic needle. Pulled sharp borosililcate glass microelectrodes (50–120 MΩ) filled with 1 M potassium acetate were passed into the brain, and the preparation was stabilized with agar. Intracellular recordings were made from RS and IB cells in layer 5 of the D2 barrel, with cell types identified through their response to depolarizing current. A 3 × 3 matrix piezoelectric stimulator was used to supply a pseudorandom sequence of whisker deflections of the PW and the eight immediate surround whiskers, allowing the receptive field of the recorded cell to be quickly mapped.

In Vitro Recording

Coronal or row traversing slices of the barrel cortex were made as described in the Supplemental Experimental Procedures. Whole-cell patch clamp recordings were made of visually identified cells in layer V of the barrel cortex, with RS and IB cells classified by their response to injected current. For mEPSC experiments a cocktail of drugs (1 μM tetrodotoxin, 10 μM picrotoxin, and 50 μM AP-V) was washed on to isolate miniature events. For morphology and depth studies, after recording the distance to the pia was measured with the patch rig manipulators and by visual measurement on the microscope. Cells were filled with biocytin to confirm their morphology post recording.

Analysis and Statistics

All analysis was performed with custom written Spike2, Matlab, and R scripts. Statistical analysis was performed with GraphPad Prism 6. All data are expressed as mean ± SEM unless otherwise stated. Data were analyzed across cohorts with one- and two-way ANOVA with Tukey's post hoc tests. All t tests were two-tailed; all alpha levels were 0.05. Linear regression and comparison of receptive fields was performed in Prism with no constraints on fit. R-squared values are quoted in the main text.

SUPPLEMENTAL INFORMATION

Supplemental Information includes four figures and Supplemental Experimental Procedures and can be found with this article online at http://dx.doi.org/10.1016/j.neuron.2015.09.025.

AUTHOR CONTRIBUTIONS

S.D.G. conceived and performed experiments, analyzed data, and wrote the manuscript. A.R. provided software and assistance with experiments. K.F. conceived the study and experiments, secured funding, analyzed data, and wrote the manuscript. S.D.G., A.R., and K.F. wrote, revised, and edited the final manuscript.

ACKNOWLEDGMENTS

We should like to thank Michael Stryker, Taro Toyoizumi, and Alison Barth for helpful criticism of the manuscript. We also thank Tim Gould for assistance with histology. This project was funded by MRC grant G0901299 and MR/N003896/1 to K.F.

Received: February 6, 2015
Revised: July 17, 2015
Accepted: September 14, 2015
Published: October 15, 2015

REFERENCES

Agmon, A., and Connors, B.W. (1989). Repetitive burst-firing neurons in the deep layers of mouse somatosensory cortex. Neurosci. Lett. *99*, 137–141.

Agmon, A., and Connors, B.W. (1992). Correlation between intrinsic firing patterns and thalamocortical synaptic responses of neurons in mouse barrel cortex. J. Neurosci. *12*, 319–329.

Chistiakova, M., Bannon, N.M., Chen, J.Y., Bazhenov, M., and Volgushev, M. (2015). Homeostatic role of heterosynaptic plasticity: models and experiments. Front. Comput. Neurosci. *9*, 89.

Connors, B.W., and Gutnick, M.J. (1990). Intrinsic firing patterns of diverse neocortical neurons. Trends Neurosci. *13*, 99–104.

Desai, N.S., Cudmore, R.H., Nelson, S.B., and Turrigiano, G.G. (2002). Critical periods for experience-dependent synaptic scaling in visual cortex. Nat. Neurosci. *5*, 783–789.

Drew, P.J., and Feldman, D.E. (2009). Intrinsic signal imaging of deprivation-induced contraction of whisker representations in rat somatosensory cortex. Cereb. Cortex *19*, 331–348.

Engert, F., and Bonhoeffer, T. (1999). Dendritic spine changes associated with hippocampal long-term synaptic plasticity. Nature *399*, 66–70.

Feldman, D.E. (2009). Synaptic mechanisms for plasticity in neocortex. Annu. Rev. Neurosci. *32*, 33–55.

Fox, K., and Wong, R.O. (2005). A comparison of experience-dependent plasticity in the visual and somatosensory systems. Neuron *48*, 465–477.

Fox, K., Glazewski, S., and Schulze, S. (2000). Plasticity and stability of somatosensory maps in thalamus and cortex. Curr. Opin. Neurobiol. *10*, 494–497.

Giese, K.P., Fedorov, N.B., Filipkowski, R.K., and Silva, A.J. (1998). Autophosphorylation at Thr286 of the alpha calcium-calmodulin kinase II in LTP and learning. Science *279*, 870–873.

Glazewski, S., and Fox, K. (1996). Time course of experience-dependent synaptic potentiation and depression in barrel cortex of adolescent rats. J. Neurophysiol. *75*, 1714–1729.

Glazewski, S., Giese, K.P., Silva, A., and Fox, K. (2000). The role of alpha-CaMKII autophosphorylation in neocortical experience-dependent plasticity. Nat. Neurosci. *3*, 911–918.

Hardingham, N., Glazewski, S., Pakhotin, P., Mizuno, K., Chapman, P.F., Giese, K.P., and Fox, K. (2003). Neocortical long-term potentiation and experience-dependent synaptic plasticity require alpha-calcium/calmodulin-dependent protein kinase II autophosphorylation. J. Neurosci. *23*, 4428–4436.

Holtmaat, A., Wilbrecht, L., Knott, G.W., Welker, E., and Svoboda, K. (2006). Experience-dependent and cell-type-specific spine growth in the neocortex. Nature *441*, 979–983.

Jacob, V., Petreanu, L., Wright, N., Svoboda, K., and Fox, K. (2012). Regular spiking and intrinsic bursting pyramidal cells show orthogonal forms of experience-dependent plasticity in layer V of barrel cortex. Neuron *73*, 391–404.

Kaneko, M., Stellwagen, D., Malenka, R.C., and Stryker, M.P. (2008). Tumor necrosis factor-alpha mediates one component of competitive, experience-dependent plasticity in developing visual cortex. Neuron *58*, 673–680.

Keck, T., Keller, G.B., Jacobsen, R.I., Eysel, U.T., Bonhoeffer, T., and Hübener, M. (2013). Synaptic scaling and homeostatic plasticity in the mouse visual cortex in vivo. Neuron *80*, 327–334.

Kirkwood, A., Silva, A., and Bear, M.F. (1997). Age-dependent decrease of synaptic plasticity in the neocortex of alphaCaMKII mutant mice. Proc. Natl. Acad. Sci. USA *94*, 3380–3383.

Krieger, P., Kuner, T., and Sakmann, B. (2007). Synaptic connections between layer 5B pyramidal neurons in mouse somatosensory cortex are independent of apical dendrite bundling. J. Neurosci. *27*, 11473–11482.

Matsuzaki, M., Honkura, N., Ellis-Davies, G.C., and Kasai, H. (2004). Structural basis of long-term potentiation in single dendritic spines. Nature *429*, 761–766.

McCormick, D.A., Connors, B.W., Lighthall, J.W., and Prince, D.A. (1985). Comparative electrophysiology of pyramidal and sparsely spiny stellate neurons of the neocortex. J. Neurophysiol. *54*, 782–806.

Ranson, A., Cheetham, C.E., Fox, K., and Sengpiel, F. (2012). Homeostatic plasticity mechanisms are required for juvenile, but not adult, ocular dominance plasticity. Proc. Natl. Acad. Sci. USA *109*, 1311–1316.

Stellwagen, D., and Malenka, R.C. (2006). Synaptic scaling mediated by glial TNF-alpha. Nature *440*, 1054–1059.

Taha, S., Hanover, J.L., Silva, A.J., and Stryker, M.P. (2002). Autophosphorylation of alphaCaMKII is required for ocular dominance plasticity. Neuron *36*, 483–491.

Toyoizumi, T., Kaneko, M., Stryker, M.P., and Miller, K.D. (2014). Modeling the dynamic interaction of Hebbian and homeostatic plasticity. Neuron *84*, 497–510.

Turrigiano, G.G. (2008). The self-tuning neuron: synaptic scaling of excitatory synapses. Cell *135*, 422–435.

Turrigiano, G.G., Leslie, K.R., Desai, N.S., Rutherford, L.C., and Nelson, S.B. (1998). Activity-dependent scaling of quantal amplitude in neocortical neurons. Nature *391*, 892–896.

Wilbrecht, L., Holtmaat, A., Wright, N., Fox, K., and Svoboda, K. (2010). Structural plasticity underlies experience-dependent functional plasticity of cortical circuits. J. Neurosci. *30*, 4927–4932.

Zenke, F., Agnes, E.J., and Gerstner, W. (2015). Diverse synaptic plasticity mechanisms orchestrated to form and retrieve memories in spiking neural networks. Nat. Commun. *6*, 6922.

Neuron, Volume *88*

Supplemental Information

Hebbian and Homeostatic Plasticity Mechanisms in Regular Spiking and Intrinsic Bursting Cells of Cortical Layer 5

Stuart David Greenhill, Adam Ranson, and Kevin Fox

Figure S1

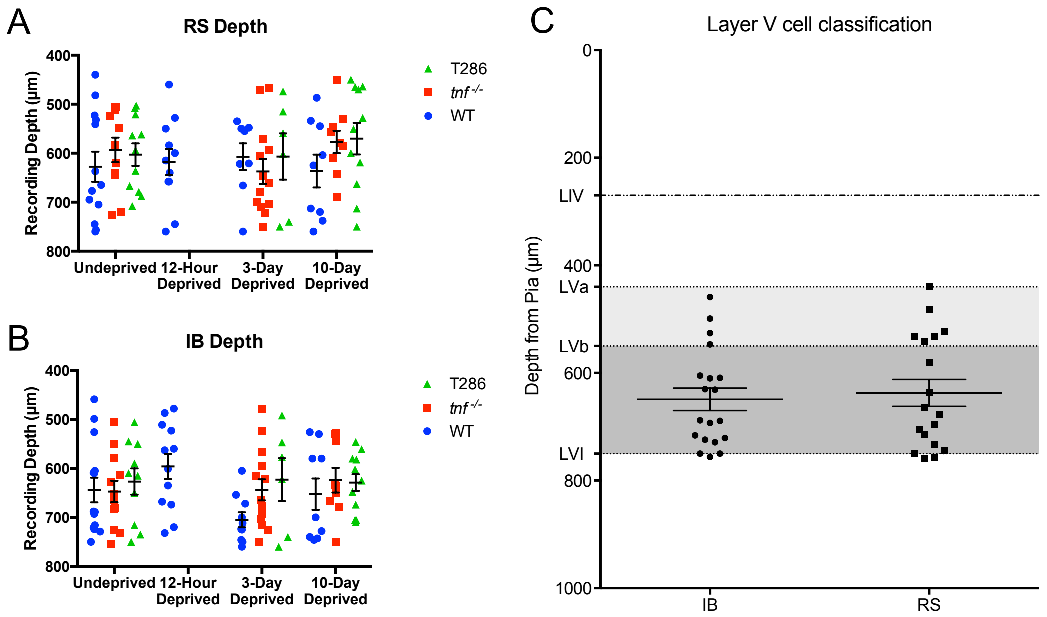

Figure S1 – Refers to Figure 1. Depth distribution of RS and IB cells recorded *in vivo* (left) and *in vitro* (right). **A** and **B.** No significant difference in depth was found between cohorts within cell types in the *in vivo* recordings (RS Deprivation: $F_{(2)}$ = 0.4219, P = 0.6572, RS Genotype: $F_{(2)}$ = 0.8028, P = s0.4516. IB Deprivation: $F_{(2)}$ = 0.6345, P = 0.5326, IB Genotype: $F_{(2)}$ = 2.063, P = 0.1333, 2-way ANOVA) either for the RS cells (top) or the IB cells (bottom). However, IB cells were encountered at a slightly greater depth than RS cells overall (644 ± 8.2 vs 607 ± 9.7, $t_{(185)}$ = 2.934, t-test). **C.** *In vitro* recordings showed that both RS and IB cell types were found throughout layer Va and Vb. No significant difference was found between the distributions of RS and IB cells in the cortical slice recordings ($t_{(35)}$ = 0.3675, mean ± sem = 688 ± 20 um for IB and 671 ± 25 um for RS, P = 0.7155, t-test). In all panels long horizontal lines indicate means and short horizontal lines indicate SEM

Figure S2

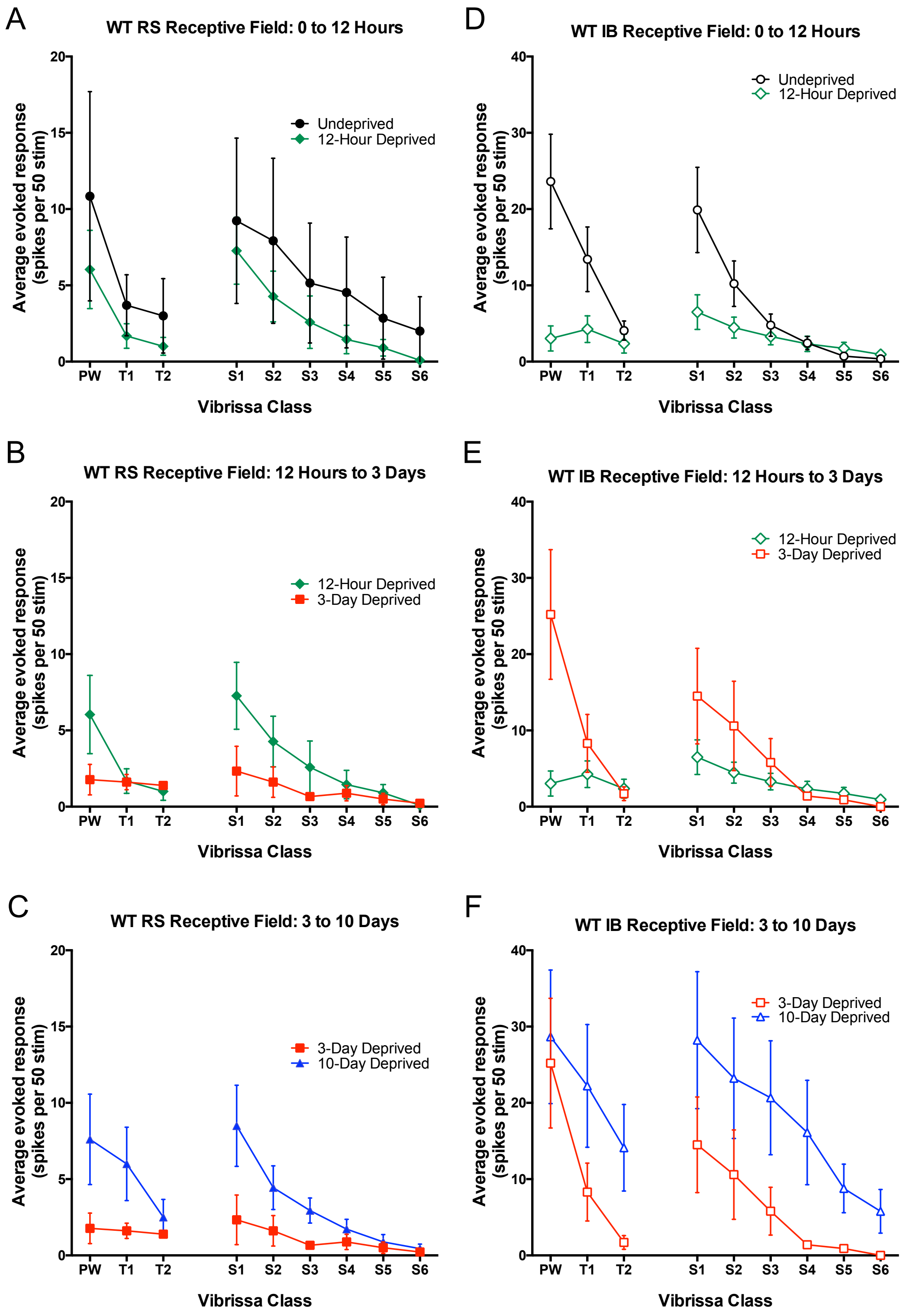

Figure S2 – Refers to Figure 2. Progression of spike responses in RS and IB cells from wild-type animals. **A.** RS cells show a slight depression between control and 12 hours deprivation (Mean response 5.47 ± 1.04 vs 2.81 ± 0.83

spikes per 50 stim) **B.** Further depression takes place between 12 hours and 3 days in RS cells (2.81 ± 0.83 vs 1.22 ± 0.23 spikes). **C.** By 10 days deprivation, RS cells have recovered towards baseline values (1.22 ± 0.23 vs 3.90 ± 0.97 spikes). **D.** IB cells show a more marked depression after 12 hours deprivation (8.83 ± 2.84 vs 3.21 ± 0.56 spikes per 50 stim). **E.** In sharp contrast to the RS cells, a recovery to baseline occurs in IB cells between 12 hours and 3 days deprivation (3.21 ± 0.56 vs 7.60 ± 2.75 spikes). **F.** IB cells, having already recovered by three days, display potentiation between 3 and 10 days deprivation (7.60 ± 2.75 vs 18.64 ± 2.69 spikes). Error bars represent SEM.

Figure S3

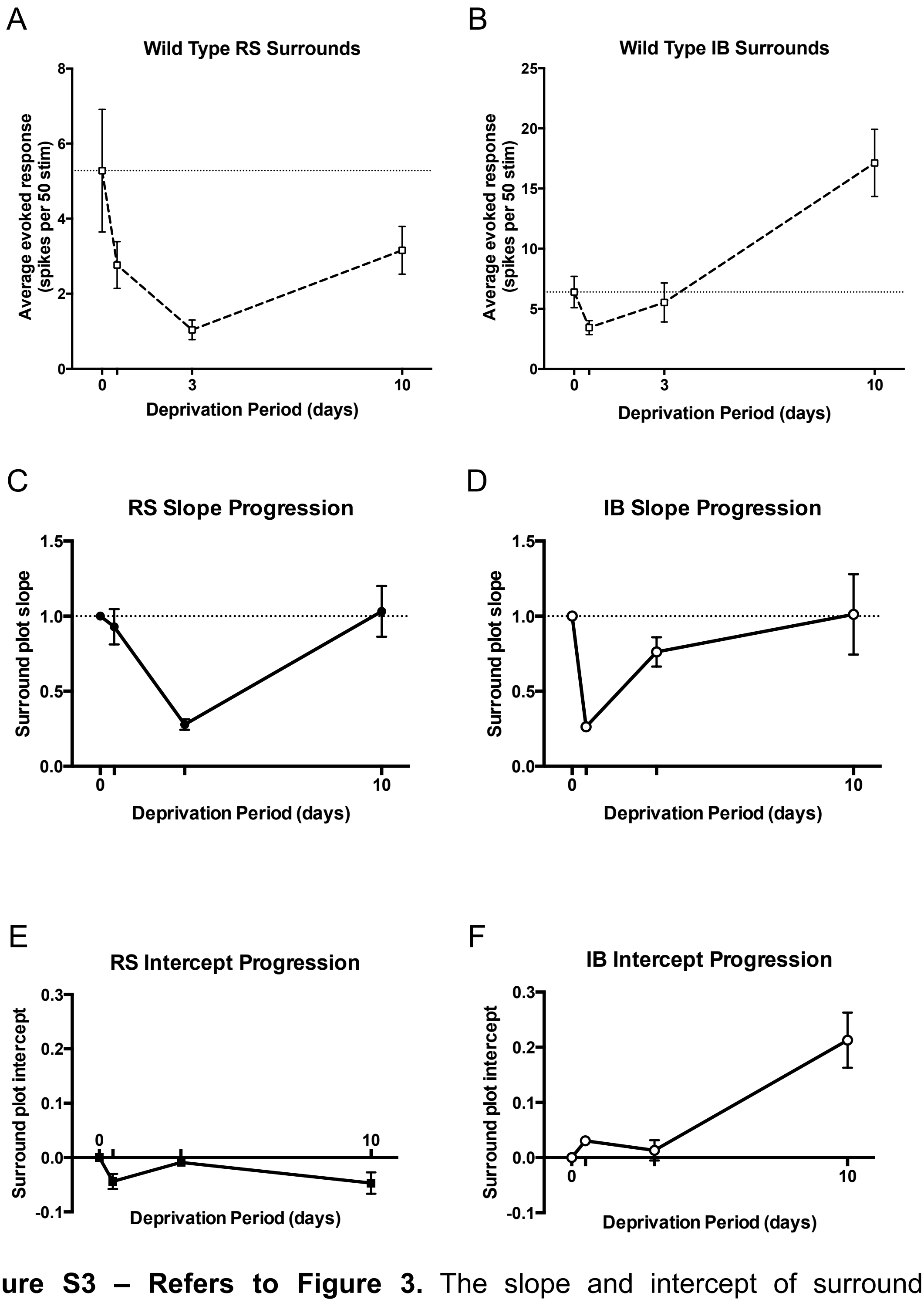

Figure S3 – Refers to Figure 3. The slope and intercept of surround responses can indicate multiplicative vs non-multiplicative plasticity shifts. **A.** Surround responses vs deprivation time in RS cells from wild-type mice. A slow depression to 3 days is followed by a partial recovery after 10 days (see also Figures 2, 3 and S2). **B.** IB surround responses show a fast depression at 12 hours, followed by recovery at 3 days and strong potentiation after 10

days deprivation (see also Figures 2, 3 and S2). **C.** The slope of the surround whisker plots displays a very similar progression to the spike responses in RS cells. A strong depression in slope at 3 days (0.28 ± 0.04 vs 1.0 for control) is followed by a return to baseline at 10 days (1.03 ± 0.17). This suggests that the control and 10 day surround receptive fields are similar but have undergone a non-multiplicative downward shift. **D.** Slope values for IB cells closely mimic the first part of the spike responses but not the latter. A rapid depression from control to 12 hours and rapid recovery to 3 days (1.0 to 0.26 ± 0.03 to 0.76 ± 0.1) is followed by a slow return to baseline as opposed to a potentiation (1.01 ± 0.27) after 10 days deprivation. **E.** RS intercepts display small downward shifts at 12 hours (-0.044 ± 0.013 12 hours, -0.047 ± 0.019 10 days). **F.** In contrast, the intercept of the IB surround plot exhibits a large positive shift between 3 and 10 days (0.013 ± 0.018 to 0.213 ± 0.050) suggesting that the potentiation seen in these cells is non-multiplicative. Error bars represent SEM.

Figure S4

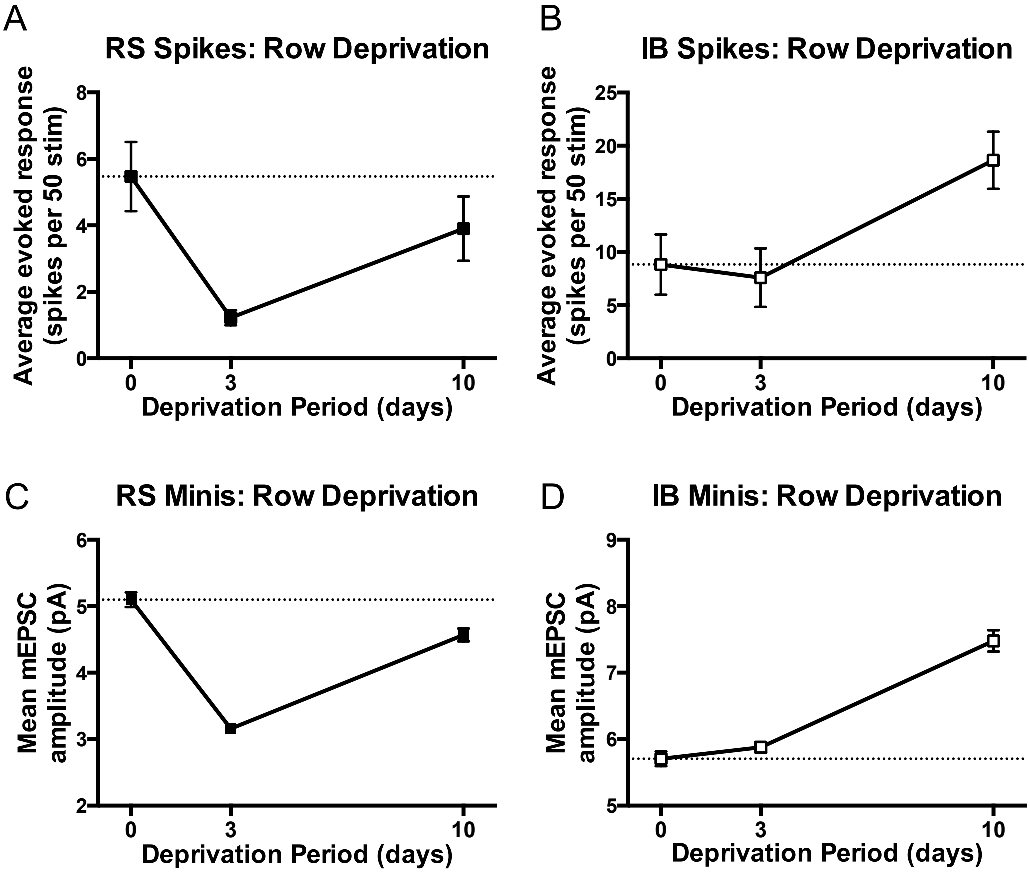

Figure S4 – Refers to Figure 5. Miniature EPSCs in row-deprived preparations *in vitro* display strong similarities to spiking responses *in vivo*. **A.** Summary of RS surround receptive field evoked spike responses – depression is followed by recovery. **B.** IB spikes, in contrast, show a strong potentiation between 3 and 10 days. **C.** RS cells in a D-row deprived *ex vivo* slice preparation: recording mEPSCs from the D-row barrels produces a progression in average mEPSC amplitude strikingly similar to that shown by the average surround receptive field spike responses. Depression at 3 days (5.10 ± 0.11 pA control vs 3.15 ± 0.04 pA 3 days) is followed by a recovery towards baseline (4.56 ± 0.10 pA) after 10 days deprivation. **D.** In contrast, the average mEPSC amplitudes of IB cells, much like their spike responses, show no depression at 3 days deprivation (5.71 ± 0.11 pA control vs 5.87 ± 0.08 pA at 3 days) but strongly potentiate after 10 days deprivation (7.48 ± 0.16 pA). Error bars represent SEM.

Supplemental Experimental Procedures

Subjects and Whisker Deprivation

All procedures were approved under the UK Animals (Scientific Procedures) Act 1986. All mice were either WT Jackson C57Bl/6J (Charles River, UK) or backcrossed into the Jackson C57Bl/6J background. A total of 63 adult male and female mice (27 C57Bl/6J wild-types, 20 TNF-α knockout (*tnf*$^{-/-}$) and 16 CamKII-T286A (T286), 6-10 weeks at recording, 213 cells total) were used for *in vivo* intracellular recording, with a further 10 wild-type mice used for histology *in vivo*. Thirty-one C57Bl/6J mice were used for *in vitro* recordings.

Subjects were lightly anaesthetised with isoflurane and had their entire right-side D-row trimmed to within 1mm of the vibrissal pad every 24-48 hours. Care was taken to trim the whiskers level with the surrounding fur but to leave enough stump for easy reattachment preceding the recording session. Deprived whiskers were replaced for recording by the corresponding whiskers from the contralateral side, attached with cyanoacrylate glue. Undeprived mice had their D-row whiskers acutely trimmed and re-attached immediately before the experiment.

In Vivo Recordings

Anaesthesia was induced with isoflurane and maintained by intraperitoneal injection of urethane (1.0 g/kg body weight) and a trace amount of acepromazine. Depth of anaesthesia was monitored by breathing rate and observation of reflexes, with supplementary urethane (0.1 g/kg) administered as necessary throughout the experiment. Body temperature was maintained at 37ºC using a thermostatically controlled heating blanket.

The location of the D-row was pinpointed before recording by intrinsic signal imaging with 700 nm light. Signals were acquired with an Optical Imaging 3001 ISI system and analysed with custom MATLAB code. Stimulus was presented as the rapid deflection of a single whisker at 5 Hz every 8 seconds using a piezoelectric wafer. At least two of D1, D2 or D3 barrels were identified and overlaid on to blood vessel patterns to gain the position and

orientation of the D-row. During electrophysiological recordings the position of the electrode was confirmed by the response to stimulus in layer IV.

After imaging a small (0.5-1 mm) craniotomy was made over the approximate location of the D2 barrel. A 30G hypodermic needle was used to open a small fleck of skull at the bottom of the craniotomy and then to resect the dura mater. A secondary craniotomy was made in posterior parietal cranium to allow for the placement of a carbon-fibre ground electrode. Borosilicate glass microelectrodes filled with 1M potassium acetate (50-120 MΩ) were passed through the small dura resection and the craniotomy covered with agar for stability. Recordings were performed with an Axoclamp 2B in current clamp mode with a manually balanced bridge and the electrode capacitance compensated. Data were acquired with a CED Micro-1401 digitiser and Spike2 software. IB and RS cells were distinguished by their response to injected current, with IB cells displaying a characteristic burst shape in response to depolarising current (Connors and Gutnick, 1990).

Whiskers were deflected using a 3x3 matrix of computer-controlled piezoelectric actuators in a custom-designed frame allowing for the resting position of the piezo to mimic the resting vibrissae position (Jacob et al., 2010; 2012). Piezo movement was controlled by a 9 whisker stimulator (CED 3901) and each piezo was regularly calibrated with a laser displacement measuring system (Micro-Epsilon). Receptive fields were quickly mapped during recording by a pseudorandom sequence of sparse noise ventrodorsal stimulations at 5Hz arranged in blocks of 10 (one of each vibrissae plus a non-stimulation event). Fifty blocks of stimulus were used for most cells, although between 15 and 100 sequences were used depending on the recording stability. Each whisker deflection was a trapezoidal stimulus (10ms rise, 10ms plateau, 10ms fall) of 300µm amplitude.

Analysis of In Vivo Recordings

Data were analysed using Spike2 software (CED) and custom R scripts. Action potentials were counted between 3-53 ms after each stimulation, with the count from the blank stimulus field subtracted as a measure of

background activity. To generate subthreshold waveforms, spikes were removed using the Wavemark tool of Spike2 and subtracting the generated spike waveforms from the original recording (Jacob et al., 2012). Whiskers were sorted from PW-T2 and S1-S6 based on their spike count alone. Subthreshold slope and latency were calculated as previously described (Jacob et al., 2012).

In Vitro Recordings

Mice (4-6 weeks old) were killed by cervical dislocation and decapitated, with the brain quickly removed and cooled in ice-cold dissection buffer (in mM: 108 choline-Cl, 3 KCl, 26 NaHCO3, 1.25 NaH2, PO4, 25 D-glucose, 3 Na-pyruvate, 1 CaCl2, 6 MgSO4, 285 mOsm, bubbled with 95% O_2 5% CO_2). Coronal slices (350µm) for depth analysis, or angled slices across the barrel rows (Finnerty et al., 1999) were cut on a vibrating microtome (Microm HM650V) and transferred to a holding chamber containing normal ACSF (in mM: 119 NaCl, 3.5 KCl, 1 NaH2PO4, 10 D-glucose, 2 CaCl2, 1 MgSO4, 300 mOsm bubbled with 95% O_2 5% CO_2). Slices were incubated at 32ºC for 45 minutes, then returned to room temperature before recording. Barrels were identified in slices under brightfield illumination and cells identified using differential interference contrast on an Olympus BX50WI microscope. Layer V was identified by distinct cellular morphology and density and whole-cell recordings were made from randomly-chosen neurones throughout both layer Va and layer Vb using borosilicate glass electrodes (4-8 MΩ) filled with a potassium-gluconate based solution (in mM: 110 K-gluconate, 10 KCl, 2 $MgCl_2$, 2 Na_2ATP, 0.03 Na_2GTP, 10 HEPES, 0.5% Biocytin, pH 7.3, 270 mOsm). For mini recordings the D-row barrel was visually identified and recordings made from within this barrel with the addition of 1µM tetrodotoxin, 10µM picrotoxin and 50µM AP-V. Recordings were made using an Axon Multiclamp 700B, digitized with a CED Micro 1401 and controlled with CED Signal software. Miniature EPSCs were analysed with Axograph software using a template-matching method.

After recordings the distance between the recording electrode and the pia was measured with Scientifica LinLab manipulator software and confirmed with a visual measurement on the microscope.

References:

Connors, B.W., and Gutnick, M.J. (1990). Intrinsic firing patterns of diverse neocortical neurons. Trends Neurosci. *13*, 99–104.

Finnerty, G.T., Roberts, L.S., and Connors, B.W. (1999). Sensory experience modifies the short-term dynamics of neocortical synapses. Nature *400*, 367–371.

Jacob, V., Estebanez, L., Le Cam, J., Tiercelin, J.-Y., Parra, P., Parésys, G., and Shulz, D.E. (2010). The Matrix: a new tool for probing the whisker-to-barrel system with natural stimuli. J. Neurosci. Methods *189*, 65–74.

Jacob, V., Petreanu, L., Wright, N., Svoboda, K., and Fox, K. (2012). Regular spiking and intrinsic bursting pyramidal cells show orthogonal forms of experience-dependent plasticity in layer V of barrel cortex. Neuron *73*, 391–404.
